# Hemoglobin's α‐Helix‐to‐β‐Sheet Transition Enables Targeted mRNA Delivery to the Lung

**DOI:** 10.1002/advs.76092

**Published:** 2026-06-12

**Authors:** Xihua Liu, Saiya Li, Guodong Wu, Shuangjian Li, Long Shuang Huang, Xiaoyang Li, Yiguo Zhao, Wei Lu, Cuixia Sun, Qin Cao, Yapeng Fang, Yiping Cao

**Affiliations:** ^1^ Department of Food Science & Engineering School of Agriculture & Biology Shanghai Jiao Tong University Shanghai China; ^2^ Shanghai Frontiers Science Center of Drug Target Identification and Delivery School of Pharmaceutical Sciences Shanghai Jiao Tong University Shanghai China; ^3^ Bio‐X Institutes Key Laboratory for the Genetics of Developmental and Neuropsychiatric Disorders Ministry of Education Shanghai Jiao Tong University Shanghai China; ^4^ School of Health Science & Engineering University of Shanghai for Science and Technology Shanghai China

**Keywords:** hemoglobin fibrils, lung targeting, mRNA delivery, platelet binding, pulmonary fibrosis

## Abstract

Effective treatment of pulmonary diseases remains constrained by the scarcity of delivery systems capable of selective tissue targeting. Herein, we report a lung‐targeting platform created through the structural repurposing of hemoglobin (Hb). Acidic heating enables a conformational shift of Hb from α‐helix to β‐sheet, leading to its self‐assembly into fibrils (HbFs). Unexpectedly, intravenously injected HbFs exhibit rapid and specific accumulation in the lungs. Cryo‐electron microscopy (cryo‐EM) structure determination revealed a fibril surface rich in positively charged residues, which facilitates two key functions: selective binding to circulating platelets via a hitchhiking mechanism for lung targeting, and efficient electrostatic complexation with mRNA. In a therapeutic application, HbFs loaded with mRNA encoding an interleukin‐11 single‐chain fragment variable (IL‐11 scFv) were administered in a murine model of bleomycin‐induced pulmonary fibrosis. The formulation achieved lung‐specific delivery with predominant uptake by pulmonary fibroblasts, enabling sustained local IL‐11 scFv expression. Consequently, treatment significantly suppressed fibroblast activation and migration, attenuated collagen deposition, restored lung function. This work establishes HbFs as a novel protein‑based vehicle for targeted mRNA delivery, leveraging natural cellular trafficking pathways to enable localized therapy for lung disorders.

## Graphical abstract

Acidic heating converts hemoglobin into β‐sheet‐rich fibrils with positively charged surfaces. These hemoglobin fibrils electrostatically complex IL‐11 scFv mRNA, selectively bind circulating platelets, and hitchhike to the lung, where they are enriched in fibroblasts. Local mRNA expression suppresses fibroblast activation, collagen deposition, and pulmonary fibrosis progression.

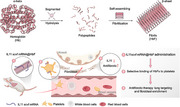

## 1 Introduction

mRNA‐based therapies represent a transformative modality in modern medicine, enabling the endogenous production of therapeutic proteins and peptides. This offers unique opportunities to drug traditionally inaccessible pathways, circumvents the risks of insertional mutagenesis associated with gene therapy, and holds promise for rapid vaccine development [1, 2, 3]. A key hurdle for their broad clinical application lies in the need for delivery systems that are safe, effective, and capable of precise tissue targeting. Lipid nanoparticles (LNPs) have established a leading role, profoundly advancing the success of mRNA vaccines. However, their application in chronic diseases requiring repeated administration remains limited. Challenges include the inherent reactogenicity of ionizable lipids [2, 3, 4, 5, 6], the potential for accelerated blood clearance mediated by anti‐polyethylene glycol (PEG) antibodies [7, 8, 9, 10], and a strong hepatic tropism that complicates the sustained delivery of mRNA to extrahepatic tissues [2, 11, 12].

Inspired by nature's own solutions, one compelling strategy is to engineer therapeutics to harness endogenous biological pathways. A clinically validated example is the class of long‐acting insulin analogs designed for reversible binding to serum albumin. This approach co‐opts the body's abundant carrier protein to create a circulating reservoir, achieving sustained pharmacokinetics while elegantly bypassing the long‐term toxicity and immunogenicity challenges frequently associated with synthetic nanocarriers [13, 14, 15, 16]. This principle motivates the broader exploration of biologically derived materials for drug delivery. Recently, protein nanofibrils, characterized by ordered β‐sheet structures, rich surface chemistry, and high biocompatibility, have shown considerable promise for the delivery of small molecules and biologics [17, 18, 19, 20, 21]. Yet, their potential for efficiently loading and delivering large nucleic acids such as mRNA remains unexplored.

**FIGURE 1:**
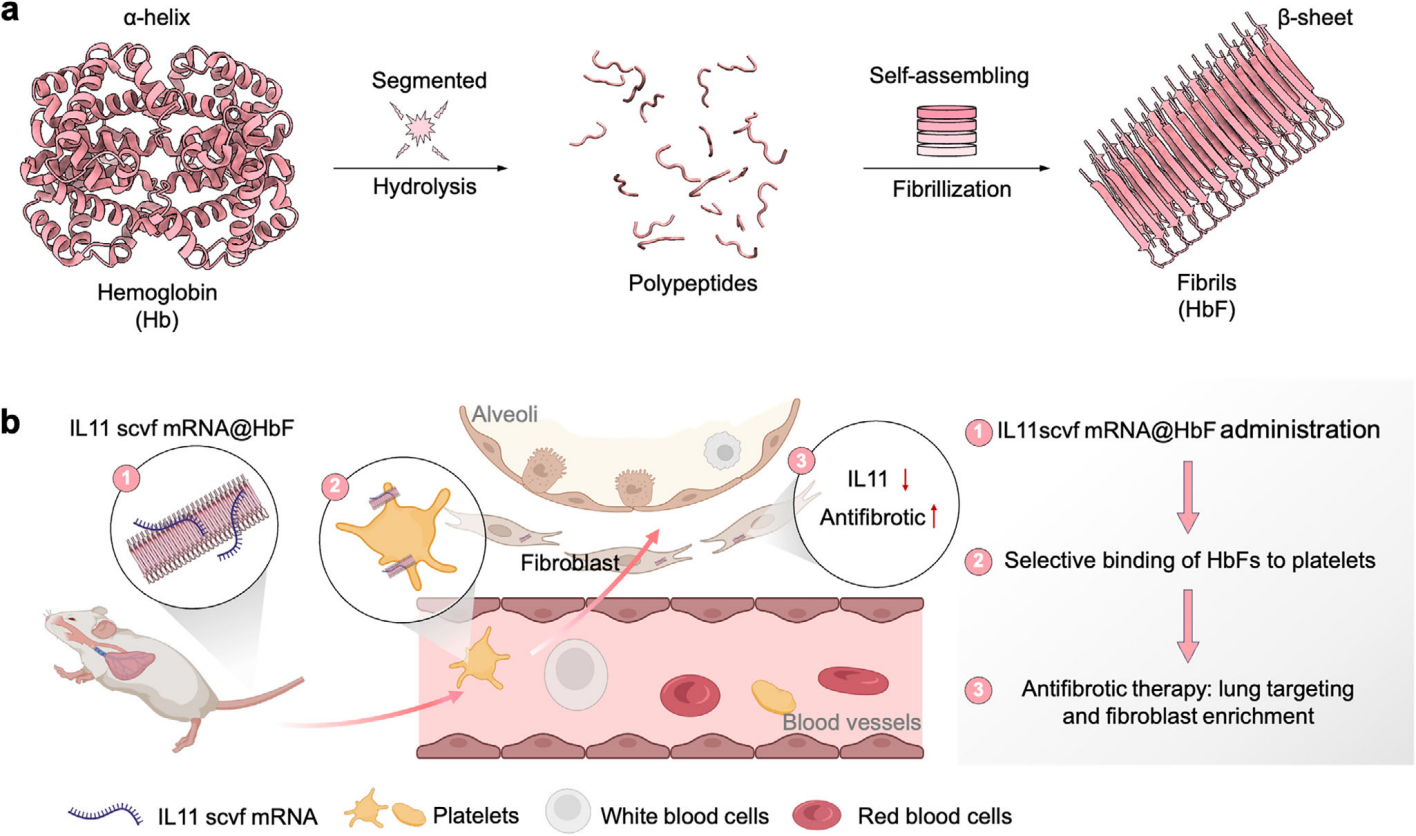
Schematic overview of hemoglobin repurposing for targeted mRNA therapy against pulmonary fibrosis. (a) Structural transformation of hemoglobin (Hb) from an α‑helical fold into β‑sheet–rich fibrils (HbFs) via acidic heating. (b) Therapeutic workflow: IL‑11 scFv mRNA is electrostatically complexed with cationic HbFs for systemic administration. Upon intravenous injection, HbFs selectively hitchhike circulating platelets, enabling lung‑targeted accumulation and subsequent fibroblast‑specific uptake. Sustained expression of the encoded IL‑11 scFv in lung fibroblasts ultimately attenuates fibrotic progression.

Herein, we report the striking observation that hemoglobin fibrils (HbFs) selectively accumulate in the lungs following intravenous administration—without the need for any engineered targeting ligands. These HbFs are prepared by subjecting hemoglobin (Hb) to acidic heating, which triggers its structural transition from a native α‐helical fold to β‐sheet‐rich assemblies (Figure 1a). To elucidate the structural basis of this distinctive tropism, we resolved the atomic structure of HbFs using cryo‐EM. Integrated structural analysis, supported by biological assays and dynamics simulations, revealed that lung targeting is mediated through a selective “platelet hitchhiking” mechanism (Figure 1b).

Building on this innate targeting property and leveraging the efficient mRNA complexation, we evaluated their therapeutic potential. Using a murine model of pulmonary fibrosis, we delivered mRNA encoding an interleukin‐11 single‐chain fragment variable (IL‐11 scFv) via HbFs. The resulting formulation demonstrated potent anti‐fibrotic efficacy both in vitro and in vivo. Overall, this work establishes a non‐LNP, protein‐based mRNA delivery platform that combines intrinsic lung targeting, high biocompatibility, and simple fabrication, offering a promising new strategy for therapies requiring repeat dosing.

## 2 Results and Discussion

### 2.1 Identifying Selective Binding of Hemoglobin Fibrils to Platelets

**FIGURE 2:**
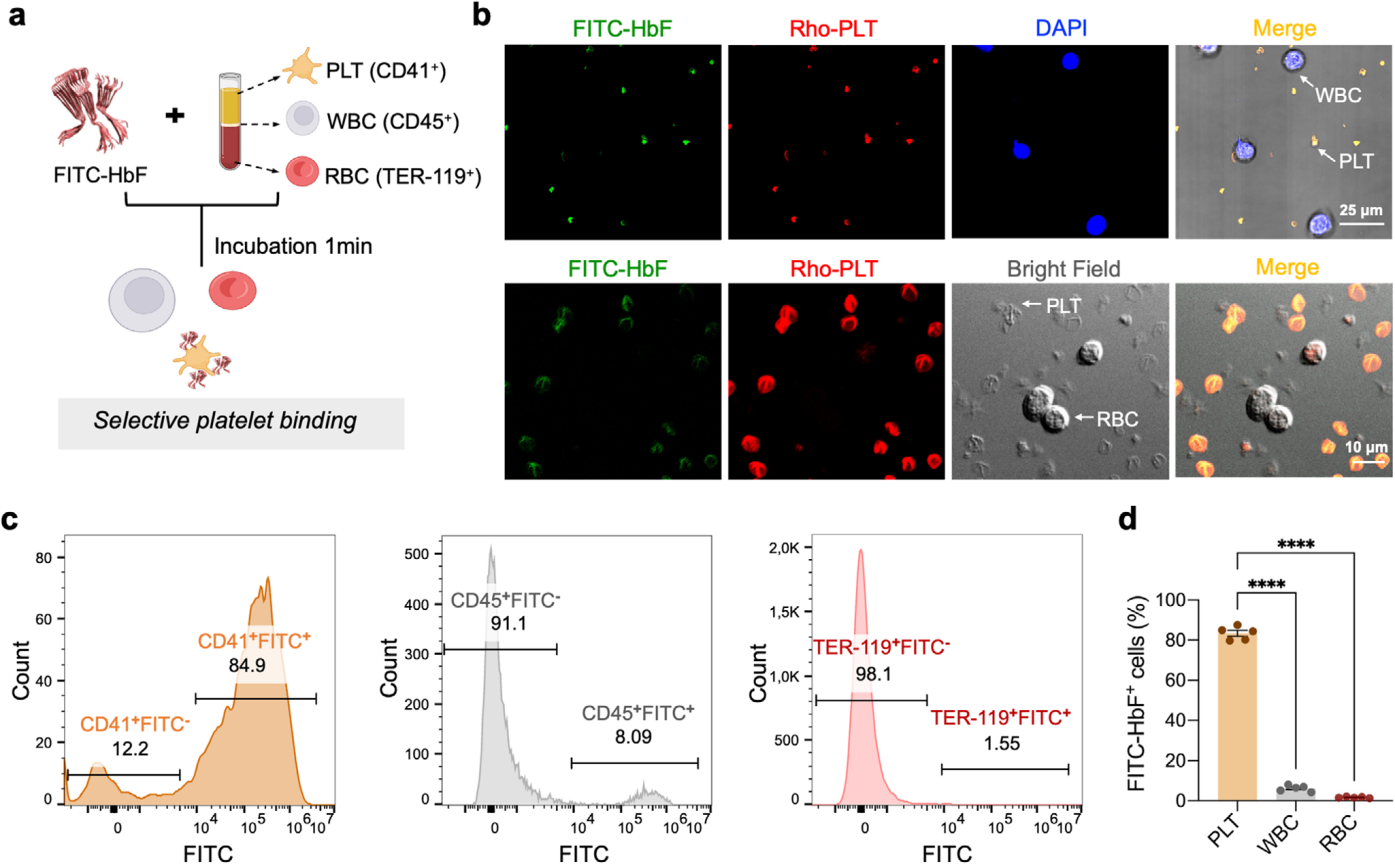
Selective binding of hemoglobin fibrils (HbFs) to platelets. (a) Schematic of HbF binding selectivity toward platelets. (b) CLSM images of blood cells incubated with FITC‐HbF (green). Platelets (PLTs) are prelabeled with Rho‐WGA (red), white blood cells (WBCs) nuclei are counterstained with DAPI (blue), and red blood cells (RBCs) are visible as disc‑shaped structures in bright field. (c) Flow cytometry analysis of cell type‐specific HbF binding. PLTs, WRCs, RBCs are identified by CD41, CD45, and TER‐119, respectively. (d) Quantification of FITC^+^ cells for each blood cell type from flow cytometry data (*n* = 5). Data are presented as mean ± SEM and were analyzed by one‐way ANOVA followed by Tukey's multiple comparisons test.

To assess the cell‐type selectivity of hemoglobin fibrils (HbFs) and probe potential specific interactions, we examined their association with major blood cell populations. Since the vast difference in natural abundance among platelets (PLTs), red blood cells (RBCs), and white blood cells (WBCs) in whole blood that could mask specific binding signals, we first isolated these cell populations from mouse blood. The isolated cells were then mixed at comparable densities to create defined mixed‐cell systems, allowing a clearer assessment of binding preference. These reconstituted cell mixtures were incubated with FITC‐labeled HbFs (FITC‐HbFs) for 1 min and subsequently analyzed by confocal laser scanning microscopy (CLSM) and flow cytometry (Figure 2). Prior to incubation, HbFs were sonicated to an average length of ∼96.7 nm to facilitate filter sterilization (Figure S1).

CLSM imaging revealed that FITC‐HbF fluorescence almost exclusively co‐localized with platelets in the mixed‐cell systems (WBCs + PLTs or RBCs + PLTs), with negligible signal detected on WBCs or RBCs (Figure 2b). Flow cytometry analysis quantitatively confirmed this pronounced selectivity. The FITC‐HbF signal was predominantly confined to the CD41^+^ platelet population, with 84.9% of platelets being FITC‐positive. In contrast, only minimal HbF binding was observed for CD45^+^ WBCs and TER‐119^+^ RBCs, with FITC‐positive cell percentages as low as 8.09% and 1.55%, respectively (Figure 2c,d). These results demonstrate that HbFs exhibit a selective, high‐affinity binding capacity for platelets over other circulating blood cells. This early preference may reflect a kinetically favored intravascular event, as platelets serve as immediate circulating interaction partners, whereas macrophage‐mediated clearance is more commonly exerted downstream by tissue‐resident phagocytes [22, 23]. In addition, the fibrillar morphology of HbF may favor rapid surface docking rather than immediate phagocytic internalization [24], and the physical properties of the platelet membrane, including its relative deformability and surface microstructure, may further support such early association [25].

### 2.2 Structural Basis for Platelet‐Selective Binding of Hemoglobin Fibrils

**FIGURE 3:**
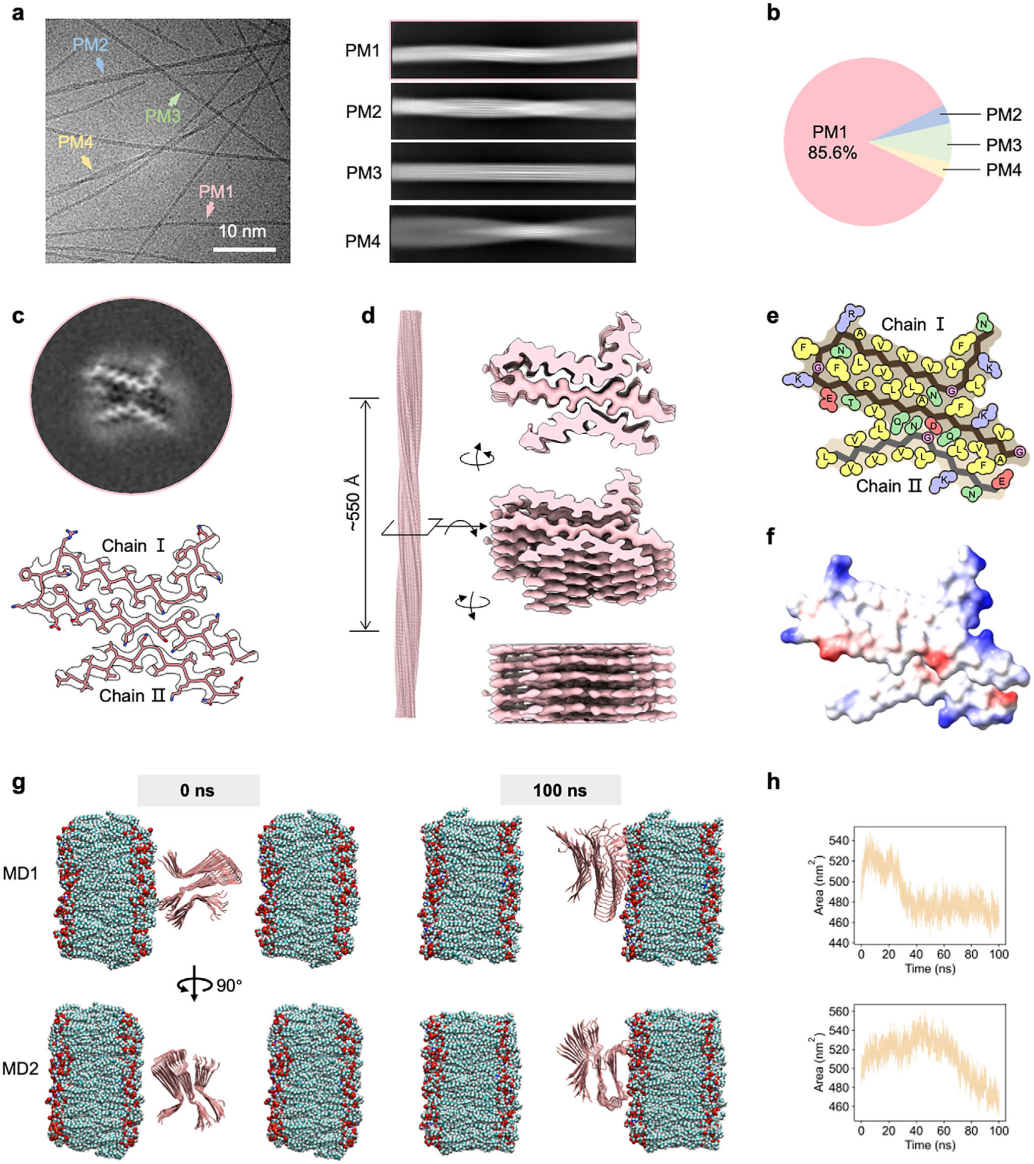
Atomic structure of HbF and structural basis for platelet binding. (a) Representative cryo‑EM micrograph of HbFs showing four morphologically distinct classes (PM1: pink, PM2: blue, PM3: green, PM4: yellow), with corresponding 2D class averages. (b) Population distribution, identifying PM1 as the predominant class. (c) Cross‐sectional view of PM1 with the atomic model fitted into the cryo‑EM density map. (d) Multiple views of PM1. (e) Space‐filling model of PM1, color‐coded by residue property (yellow: hydrophobic; green: polar; red: acidic; blue: basic; pink: glycine). (f) Surface electrostatic potential of PM1, mapped from negative (red) to positive (blue). (g) MD simulation systems illustrating different initial orientations of HbFs relative to the platelet membrane; all converged to a similar binding mode after 100 ns. Other two independent stimulations are shown in Figure S4. (h) Evolution of the solvent accessible surface area at the fibril‐membrane interface over the 100 ns simulation time.

To elucidate the structural mechanism underlying the selective platelet binding of HbFs, we determined their atomic structure using cryo‑EM (detailed workflow in Figure S2). 2D classification revealed four distinct fibrillar morphologies (Figure 3a). Quantitative analysis of 2430 micrographs indicated that PM1 was the predominant species, constituting 85.6% of the total population (Figure 3b). The reconstructed cryo‑EM map of PM1 achieved an overall resolution of 3.3 Å (Figure S3), enabling reliable atomic model building. As shown in Figure 3c, the atomic model exhibited an excellent fit to the map density. Notably, PM1 was formed exclusively by peptides derived from Hb beta subunit (UniProt ID: P02070), despite the use of full‐length Hb as the starting material.

Structural analysis revealed that PM1 surface is enriched with positively charged residues, including Lys103, Arg115, Lys119, and Lys131 in chain I, and Lys103 in chain II, alongside a limited number of negatively charged residues such as Glu120 (chain I) and Glu100 (chain II) (Figure 3e,f). This net positive surface charge suggests a strong potential for electrostatic interactions with negatively charged molecules—a property we later leveraged for mRNA complexation and delivery.

To investigate the structural basis for platelet binding, we performed molecular dynamics (MD) simulations using the resolved PM1 structure and a modeled platelet membrane bilayer. Four independent simulation systems were initiated with the fibril placed in distinct orientations relative to the membrane (Figure 3g and Figure S4). All systems converged to a consistent binding mode within 100 ns, in which the Asn101‐Arg115 segment of chain I formed the primary binding interface. Analysis of the solvent accessible surface area (SASA) at the fibril‑membrane interface showed a rapid decrease from an initial ∼540 nm^2^ to a stable plateau around 460 nm^2^ across all replicates (Figure 3h and Figure S4), confirming the establishment of a stable interaction.

### 2.3 Lung Targeting and Fibroblast‐Selective Uptake of Platelet‐Hitchhiking HbFs

**FIGURE 4:**
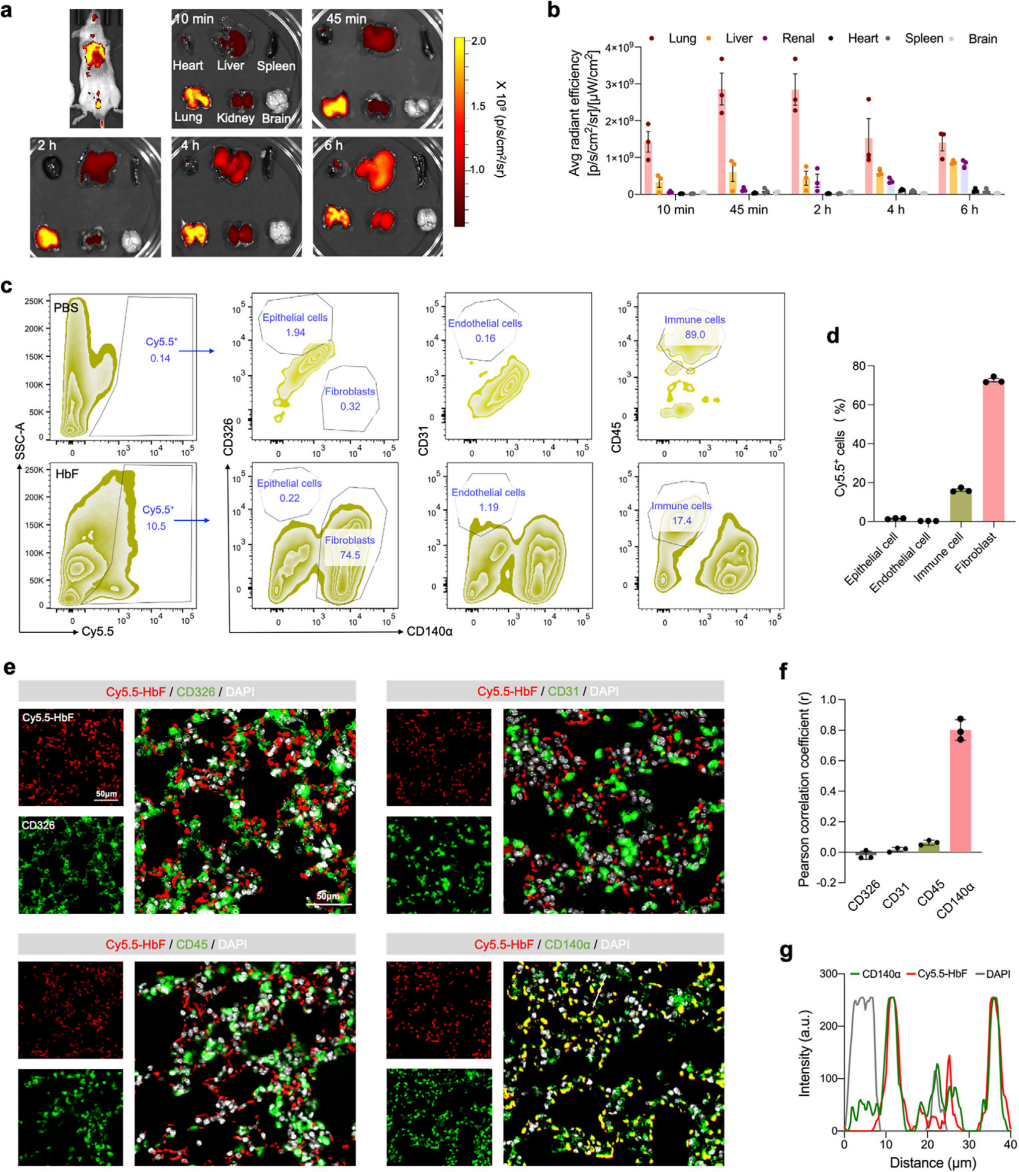
Lung targeting and fibroblast‐selective uptake of platelet‐hitchhiking HbFs. (a) Ex vivo fluorescence images and (b) quantification of Cy5.5 radiant efficiency in major organs at indicated time points after intravenous injection of Cy5.5‑HbFs (2 mg/mL, 200 µL/mouse). (c, d) Flow cytometry contour plots of lung single‐cell suspensions from PBS‐ or HbF‐treated mice: (c) gating strategy for epithelial cells (CD326^+^), fibroblasts (CD140α^+^), endothelial cells (CD31^+^), and immune cells (CD45^+^); (d) percentages of Cy5.5^+^ cells within each lung cell subset. (e) Representative immunofluorescence images of lung sections showing Cy5.5‐HbFs (red) and cell‑type markers (green: CD326, CD31, CD45 or CD140α); nuclei are stained with DAPI (shown as white). (f) Pearson correlation coefficients between Cy5.5‐HbF signal and each cellular marker. (g) Representative line‐scan intensity profiles along the dashed line in the CD140a panel. Data are presented as mean ± SEM (*n* = 3).

Informed by recent evidence highlighting the pulmonary microvasculature as a primary site of platelet production [26, 27], we posited that platelet‐hitchhiking HbFs would exhibit pronounced lung tropism in vivo. Following intravenous injection of Cy5.5‑HbFs (2 mg/mL, 200 µL/mouse), we observed rapid and specific accumulation in the lungs, with significant enrichment detectable within ∼10 min and maintained for up to 6 h (Figure 4a,b). In contrast, Cy5.5‑labeled native hemoglobin distributed predominantly to the liver, with minimal lung preference (Figure S5), confirming that the lung‐targeting capability arises specifically from the β‐sheet fibrillar structure of HbFs.

To identify the lung cellular populations responsible for this uptake, we performed flow cytometry on enzymatically digested lung tissue, using CD140a, CD326, CD31, and CD45 to identify fibroblasts, epithelial cells, endothelial cells, and immune cells, respectively [28]. Uptake was overwhelmingly concentrated in fibroblasts: 74.5% of CD140a^+^ cells were Cy5.5^+^. In comparison, only 17.4% of CD45^+^ immune cells were positive, while uptake in CD326^+^ epithelial and CD31^+^ endothelial cells was nearly undetectable (Figure 4c,d; gating strategy shown in Figure S6). Immunofluorescence analysis of lung sections corroborated these findings, demonstrating strong colocalization of Cy5.5‐HbFs with CD140a^+^ fibroblasts (Pearson correlation coefficient, r ≈ 0.80), but not with CD45^+^ immune cells (r ≈ 0.063), CD326^+^ epithelial cells (r ≈ ‐0.019), or CD31^+^ endothelial cells (r ≈ 0.017) (Figure 4e,g).

This distinct cellular distribution aligns with the known architecture of the pulmonary barrier. The lung microvascular endothelium is rich in caveolae, which are known to mediate the trans‐endothelial transport of macromolecules under physiological conditions [29, 30, 31]. Resident fibroblasts, situated in the perivascular interstitium, thus represent a logical final reservoir for extravasated HbFs. Together, these data demonstrate that platelet‐hitchhiking HbFs rapidly home to the lung upon systemic administration and are preferentially internalized by pulmonary fibroblasts, establishing a clear foundation for their use as a fibroblast‐targeted delivery platform.

### 2.4 HbF Enables Efficient and Sustained mRNA Expression in the Lung

**FIGURE 5:**
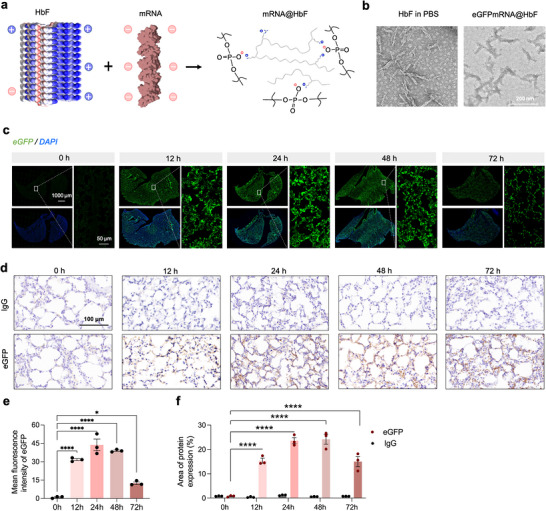
Efficient and sustained mRNA delivery to the lung mediated by HbFs. (a) Schematic of electrostatic complexation between positively charged HbFs and negatively charged mRNA. (b) TEM images of HbF alone (left) and eGFP mRNA@HbF (right). (c) Representative eGFP fluorescence images of lungs and (e) quantification of mean eGFP intensity at the indicated time points after intravenous injection of eGFP mRNA@HbF (10 µg mRNA/mouse); eGFP signal is shown in green and nuclei are stained with DAPI (blue). (d) Representative immunohistochemical staining of lung sections for eGFP (IgG as negative control) and (f) quantification of eGFP‑positive lung area at indicated time points after eGFP mRNA@HbF injection (brown, positive staining; nuclei counterstained with hematoxylin). Data are presented as mean ± SEM (*n* = 3) and were analyzed by one‐way ANOVA followed by Tukey's multiple comparisons test. Significance levels: *p ≤ 0.05, ****p ≤ 0.0001.

Having established the platelet‐hitchhiking and lung fibroblast‐targeting properties of HbFs, we next evaluated their capacity for in vivo mRNA delivery. Using eGFP‐encoding mRNA as a reporter, we complexed it electrostatically with cationic HbFs to form eGFP mRNA@HbF complex (Figure 5a). Successful complexation was confirmed by a shift in zeta potential (Figure S7a) and visualization via TEM (Figure 5b), and colocalization between HbFs and eGFP mRNA in cells further validated complex stability (r ≈ 0.77; Figure S7b). Notably, comparable electrophoretic‐profile and potential were retained after storage at 4°C for 1 month, indicating good stability under the tested condition (Figure S13a,b).

Following intravenous injection of eGFP mRNA@HbF (10 µg mRNA/mouse), strong eGFP fluorescence was detected in the lungs at 12 h, peaked around 24 h, and remained for 48–72 h before gradually declining (Figure 5c,e). In contrast, negligible eGFP signal was observed in the liver or kidney (Figure S8), and free eGFP mRNA produced almost no lung fluorescence (Figure S9). Immunohistochemistry further confirmed efficient and sustained eGFP protein expression exclusively in lung sections from treated mice (Figure 5d,f; Figures S10 and S11). Together, these results suggest that the HbF carrier is required for efficient in vivo mRNA delivery and expression in the lung, while functional protein expression remains largely restricted to this target tissue. Accordingly, the delayed Cy5.5 fluorescence observed in the liver and kidney in Figure 4a is more likely to reflect carrier‐associated processing and clearance than functional off‐target expression.

In primary mouse lung fibroblasts (MLFs), eGFP mRNA@HbF induced eGFP expression with efficiency comparable to the commercial reagent Lipofectamine 2000 (Figure S12), and mRNA@HbFs stored for 1 month retained comparable transfection efficiency (Figure S13c,d). Similar transfection results were also obtained in NIH/3T3 and MEF cell lines with the freshly prepared formulation (Figure S14). Western blot analysis confirmed sustained eGFP production in MLFs, with protein detectable for at least 72 h post‑treatment (Figure S15). Notably, these in vitro transfection results were obtained in the absence of platelets, confirming that the internalization of HbF‐mRNA complexes by target cells occurs through platelet‑independent mechanisms. Combined with the in vivo platelet‑hitchhiking and fibroblast‑selective uptake data (Figure 4), this distinction suggests that platelets primarily serve as intravascular pulmonary delivery carriers, while the subsequent cellular uptake by fibroblasts likely follows local disengagement of HbF in the pulmonary capillary bed, analogous to the previously reported RBC‑hitchhiking paradigm [32, 33].

These findings further indicate that HbF facilitates endosomal escape of eGFP mRNA, enabling highly efficient mRNA expression. Consistently, eGFP mRNA@HbF initially co‐localized with lysosomes (Lyso‐Tracker) and late endosomes/lysosomes (Rab7) at 1 h, but this association decreased significantly by 3–5 h, indicating progressive escape from endolysosomal compartments (Figure S16). This behavior is attributed to the surface‑exposed lysine and arginine residues on HbF, which increase its net positive charge under acidic lysosomal conditions (Figure S17)—a feature known to promote endosomal membrane disruption [34, 35]. Together, these data demonstrate that HbF can effectively leverage the fibroblast‑targeting properties to achieve potent, lung‑restricted, and sustained mRNA expression in vivo.

### 2.5 IL‐11 scFv mRNA@HbF Attenuates Fibrotic Activation and Migration of MLFs

Interleukin‐11 (IL‐11) is a validated therapeutic target in pulmonary fibrosis [36, 37, 38]. Inhibition of its signaling pathway has been shown to attenuate or reverse lung fibrosis in multiple animal models, and antibodies targeting IL‑11 or its receptor have entered early‑stage clinical development [36, 37, 38, 39]. Leveraging the ability of HbF to enable fibroblast‐targeted and sustained mRNA delivery (Figure 5), we engineered HbF to deliver mRNA encoding an IL‐11 single chain fragment variable (IL‐11 scFv), generating the complex IL‐11 scFv mRNA@HbF. Its antifibrotic efficacy was then evaluated in primary mouse lung fibroblasts (MLFs) stimulated with the profibrotic cytokine TGF‑β1, which is known to enable the fibroblast‐to‐myofibroblast transition through IL‑11 induction [36, 38, 40].

**FIGURE 6:**
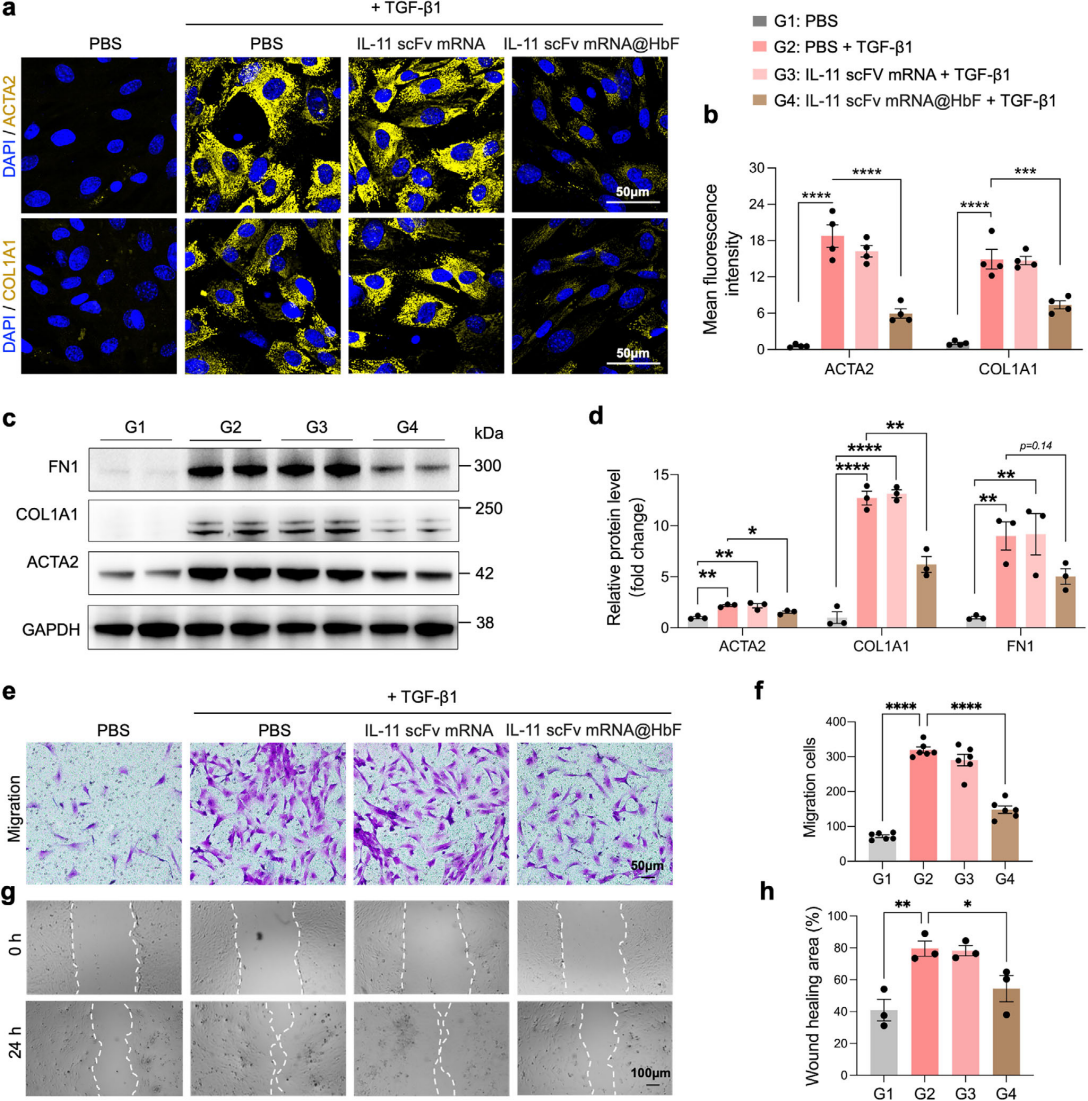
IL‐11 scFv mRNA@HbF inhibits fibrotic activation and migration of primary mouse lung fibroblasts (MLFs). MLFs were treated with TGF‐β1 (10 ng/mL) for 24 h to induce fibrotic activation, alongside PBS (G2), IL‐11 scFv mRNA (0.5 µg/mL; G3), or IL‐11 scFv mRNA@HbF (equivalent mRNA dose; G4). MLFs treated with PBS only were used as a negative control (G1). (a, b) Representative immunofluorescence images of ACTA2 and COL1A1 expression in MLFs, with corresponding quantitative analysis. (c, d) Western blot analysis and corresponding densitometry of ACTA2, COL1A1 and FN1 protein levels. (e, f) Transwell migration assay: (e) representative crystal violet‐stained images and (f) quantification of migrated cells per field. (g, h) Wound‐healing assay: (g) representative images and (h) quantification of wound closure expressed as the percentage of wound area at 24 h relative to 0 h. Data are presented as mean ± SEM (*n* ≥3) and were analyzed by one‐way ANOVA followed by Tukey's multiple comparisons test. Significance levels: *p ≤ 0.05, **p ≤ 0.01, ***p ≤ 0.001, ****p ≤ 0.0001.

Immunofluorescence staining confirmed that stimulation with TGF‐β1 (10 ng/mL, 24 h) significantly upregulated the expression of myofibroblast markers actin alpha 2 (ACTA2) and collagen I alpha 1 chain (COL1A1) in MLFs. This upregulation was markedly suppressed by IL‐11 scFv mRNA@HbF, whereas free IL‐11 scFv mRNA failed to achieve this effect (Figure 6a,b). Western blot analysis (Figure 6c,d) further demonstrated that IL‐11 scFv mRNA@HbF can significantly downregulate the TGF‐β1‐induced overexpression of ACTA2 and COL1A1, as well as fibronectin 1 (FN1).

Because the fibroblast‐to‐myofibroblast transition is typically associated with increased migratory capacity [41, 42], we performed Transwell migration assay to evaluate the impact of IL‐11 scFv mRNA@HbF on MLF migration. TGF‑β1 stimulation significantly increased the number of migrated MLFs, and this pro‑migratory effect was substantially inhibited by IL‑11 scFv mRNA@HbF (Figure 6e,f). Consistent with these results, wound‐healing assay showed that IL‐11 scFv mRNA@HbF significantly suppressed TGF‐β1‐induced wound contraction (Figure 6g,h). Together, these findings suggest that IL‐11 scFv mRNA@HbF can effectively inhibit fibroblast activation and migration—two critical processes in the development of fibrotic lesions [41, 42]—highlighting its potential as a therapeutic strategy for pulmonary fibrosis.

### 2.6 IL‐11 scFv mRNA@HbF Attenuates Bleomycin‐induced Pulmonary Fibrosis

**FIGURE 7:**
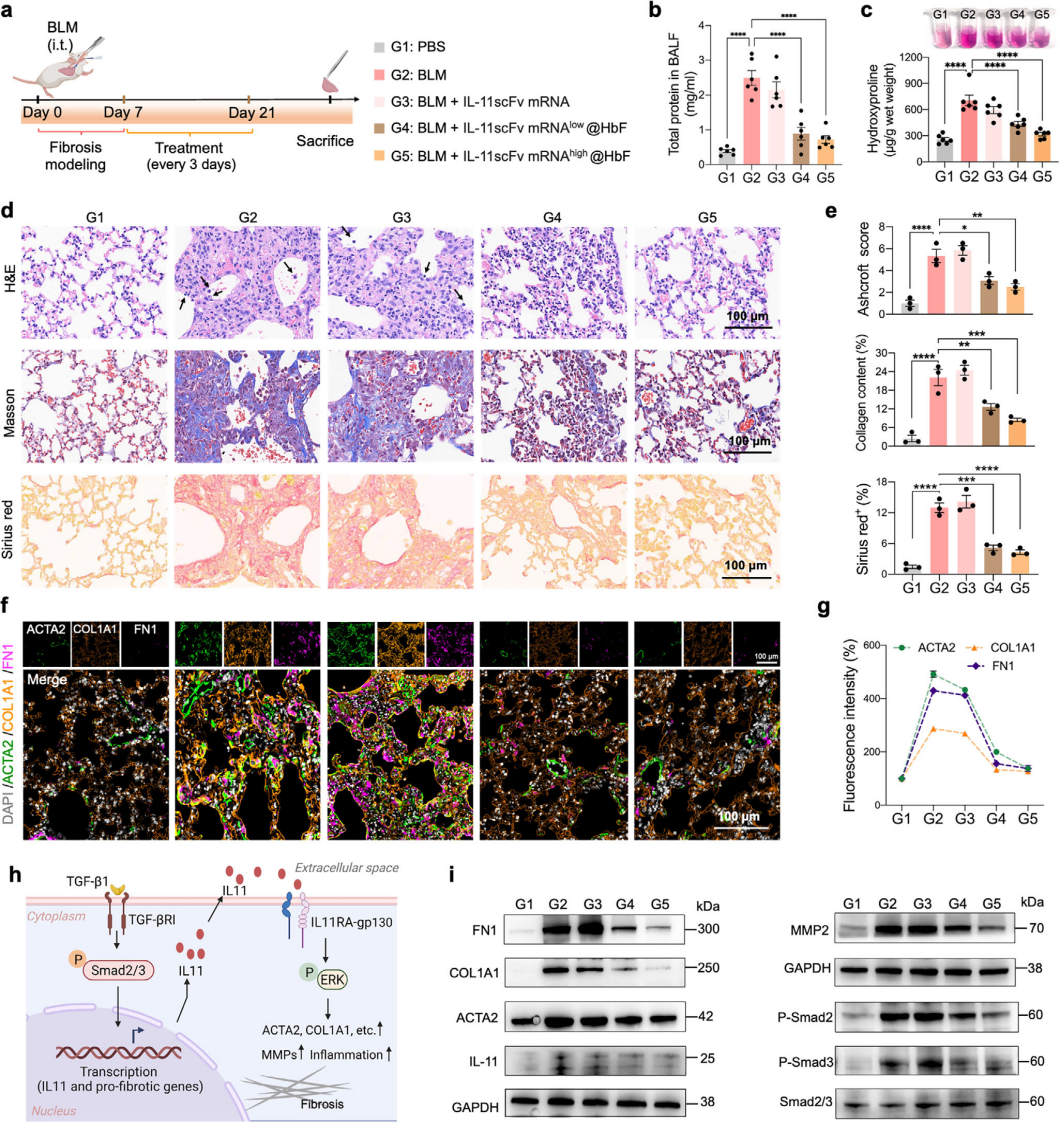
IL‐11 scFv mRNA@HbF attenuates bleomycin‐induced lung fibrosis in mice. (a) Experimental design of the animal study. Groups: G1, PBS control; G2, BLM only; G3, BLM + IL‐11 scFv mRNA (15 µg/mouse); G4, BLM+ IL‑11 scFv mRNA@HbF low dose (10 µg/mouse); G5, BLM+ IL‑11 scFv mRNA@HbF high dose (15 µg/mouse). (b) Total protein content in BALF, n = 6. (c) Lung hydroxyproline content, *n* = 6. (d, e) Representative lung sections stained with H&E, Masson's trichrome and Sirius red, with corresponding quantitative analysis; arrows in H&E images indicate inflammatory cell infiltration; n = 3. (f, g) Representative multiplex immunofluorescence images of lung sections stained for ACTA2 (green), COL1A1 (yellow), FN1 (magenta) and nuclei (DAPI, blue), with quantification of fluorescence intensity for each marker, n = 3. (h) Schematic of the TGF‐β1/IL‐11 signaling axis in fibroblast activation [38]. (i) Western blot analysis of ACTA2, COL1A1, FN1, IL‐11, phospho‐Smad2/3, total Smad2/3, and MMP2 in lung tissues (see Figure S19 for quantification, n = 3). Data are presented as mean ± SEM and were analyzed by one‐way ANOVA followed by Tukey's multiple comparisons test. Significance levels: *p ≤ 0.05, **p ≤ 0.01, ***p ≤ 0.001, ****p ≤ 0.0001.

To evaluate the in vivo antifibrotic efficacy of IL‐11 scFv mRNA@HbF, we utilized a well‐established mouse model of pulmonary fibrosis induced by a single intratracheal dose of bleomycin (BLM) [36]. Mice were allowed to progress to day 7 post‐BLM, corresponding to the established early fibrotic phase commonly used for therapeutic intervention [36]. Beginning on day 7, mice received intravenous injections every 3 days of either PBS (G2), free IL‐11 scFv mRNA (15 µg/mouse; G3), or IL‐11 scFv mRNA@HbF at low (10 µg/mouse; G4) or high (15 µg/mouse; G5) doses until the experimental endpoint on day 21 (Figure 7a). Mice receiving PBS without BLM treatment served as the negative control (G1). Body weight was recorded every 2 days throughout the study (Figure S18).

As shown in Figure 7b,c, BLM injury significantly increased the total protein content in bronchoalveolar lavage fluid (BALF), indicative of alveolar‐capillary leakage, and elevated lung hydroxyproline content, reflecting collagen accumulation. This damage was reversed by IL‐11 scFv mRNA@HbF treatment in a dose‐dependent manner, whereas free IL‐11 scFv mRNA was ineffective.

Histopathological analysis further supported the antifibrotic effects (Figure 7d,e). H&E staining revealed that BLM‐induced alveolar septal thickening, architectural distortion and inflammatory cell infiltration were markedly alleviated by IL‐11 scFv mRNA@HbF treatment, with greater recovery at the higher dose. Masson's trichrome and Sirius red staining demonstrated that IL‐11 scFv mRNA@HbF dose‐dependently reduced interstitial collagen deposition and fibrotic septa formation. These findings align with previous reports that blocking IL‐11 signaling can inhibit or reverse BLM‐induced pulmonary fibrosis [38, 39, 43].

In pulmonary fibrosis, TGF‐β1 upregulates IL‐11 expression via Smad2/3‑mediated transcriptional activation [38]. Secreted IL‐11 then engages the IL‑11RA‐gp130 receptor, activating downstream ERK and JAK/STAT signaling [38], which amplifies the expression of myofibroblast markers (ACTA2, COL1A1, FN1) and matrix‑remodeling enzymes such as MMPs (Figure 7h). To validate the engagement of this axis in our model, we performed multiplex immunofluorescence and western blot analyses. As shown in Figure 7f,g, BLM‐induced increase in ACTA2, COL1A1, and FN1 expression was substantially suppressed by IL‑11 scFv mRNA@HbF treatment. Western blotting further confirmed that IL‑11 scFv mRNA@HbF markedly reduced the protein levels of these fibrotic markers, whereas free IL‑11 scFv mRNA showed no effect (Figure 7i, Figure S19). Notably, IL‑11 scFv mRNA@HbF also lowered the levels of IL‑11 itself and its downstream effector MMP2. By disrupting this positive feedback loop, the treatment consequently attenuated the activation of upstream phospho‑Smad2/3, demonstrating effective in vivo blockade of the IL‐11‐driven profibrotic axis (Figure 7i, Figure S19). Although the separation between the low‐dose and high‐dose groups was modest, the overall pattern suggests that the lower dose already achieved substantial efficacy, while the higher dose provided only limited additional benefit within the tested dose range.

**FIGURE 8:**
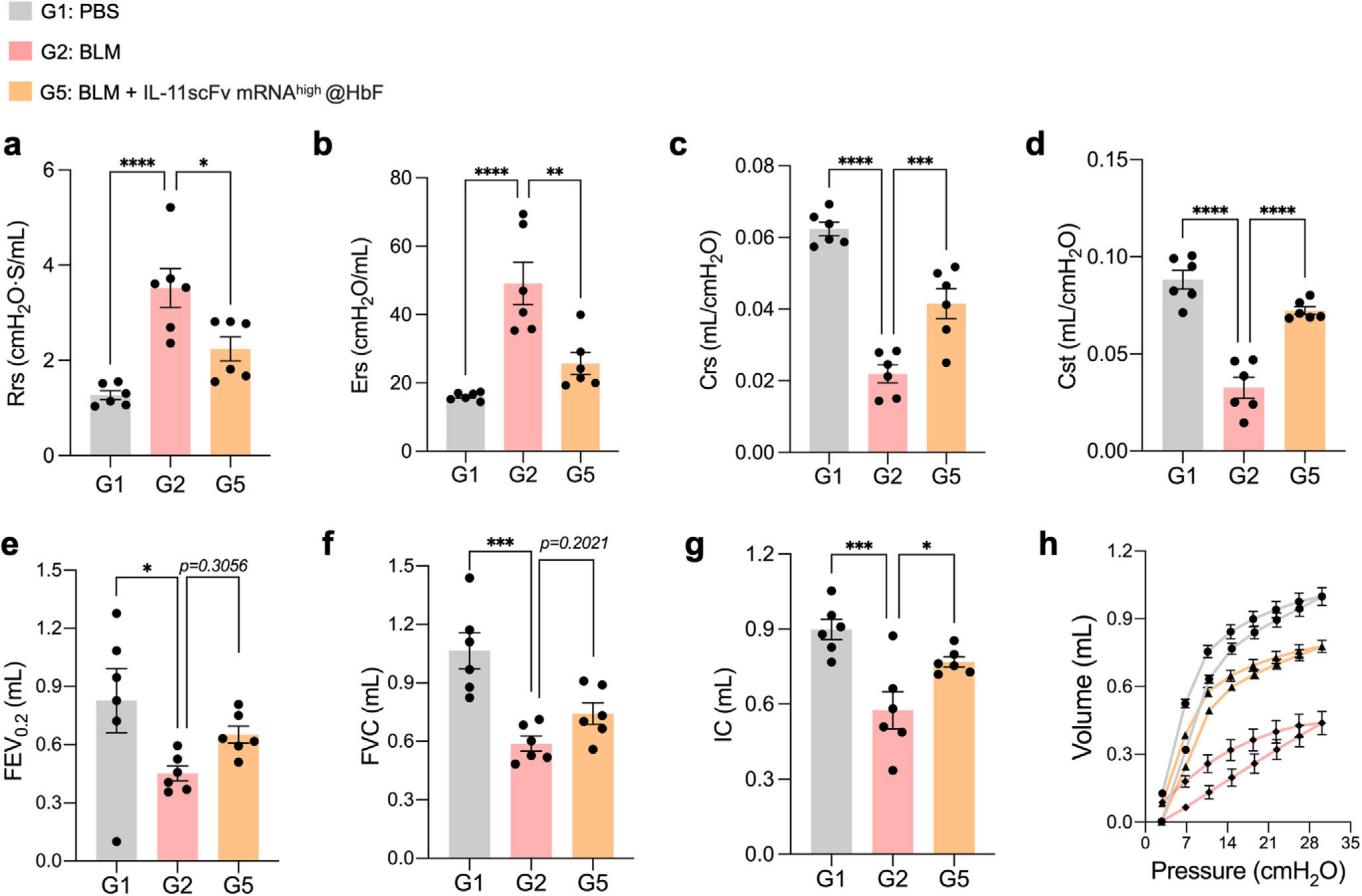
IL‐11 scFv mRNA@HbF restores pulmonary function for bleomycin‐induced fibrosis. Lung function was assessed by forced oscillation technique on day 21. Groups are as defined in Figure 7a. Parameters shown: (a) respiratory system resistance (Rrs), (b) elastance (Ers), (c) dynamic compliance (Crs), (d) static compliance (Cst), (e) forced expiratory volume in 0.2 s (FEV_0_._2_), (f) forced vital capacity (FVC), and (g) inspiratory capacity (IC). (h) Quasi‐static pressure‐volume (P‐V) curves. Data are presented as mean ± SEM (*n* = 6) and were analyzed by one‐way ANOVA followed by Tukey's multiple comparisons test. Significance levels: *p ≤ 0.05, **p ≤ 0.01, ***p ≤ 0.001, ****p ≤ 0.0001.

Consistent with the histological and molecular improvements, pulmonary function assessed by forced oscillation technique showed clear recovery following IL‐11 scFv mRNA@HbF treatment (Figure 8). Fibrotic model mice (G2) exhibited significantly elevated respiratory system resistance (Rrs) and elastance (Ers), indicating increased lung stiffness and reduced compliance [39, 43], both of which were effectively reversed by IL‐11 scFv mRNA@HbF treatment (G5) (Figure 8a,b). The therapy also ameliorated deficits in key volume and compliance parameters, including dynamic compliance (Crs), static compliance (Cst), forced vital capacity (FVC), forced expiratory volume in 0.2 s (FEV_0_._2_), and inspiratory capacity (IC), with particularly notable recovery in compliance and inspiratory volumes (Figure 8c–g). Quasi‐static pressure‐volume (P‐V) loop analysis corroborated these findings (Figure 8h). Treatment with IL‐11 scFv mRNA@HbF shifted the P‐V loop upward toward the normal range, demonstrating restored pulmonary mechanics.

## 3 Conclusion

In summary, this study demonstrates that a simple α‐helix‐to‐β‐sheet conformational transition of hemoglobin can be harnessed to generate a bio‐derived nanomaterial with an intrinsic ability to target the lung. We have delineated a coherent delivery mechanism: the resulting hemoglobin fibrils (HbFs) selectively hitchhike on circulating platelets, co‐opting their physiological trafficking to achieve efficient pulmonary accumulation. Structural analysis further revealed a positively charged fibril surface that mediates both this cellular hitchhiking and the stable electrostatic complexation of mRNA. Capitalizing on this targeted delivery, we showed that HbFs can effectively transport therapeutic mRNA, specifically delivering an IL‐11‐neutralizing agent to lung fibroblasts. In an established model of pulmonary fibrosis, this strategy attenuated core pathological features—including fibroblast‐to‐myofibroblast transition, collagen deposition, and impaired lung function—thereby confirming its therapeutic efficacy.

This study presents several key advances. First, it demonstrates a simple route to convert hemoglobin—the most abundant protein in blood—into programmable fibrils, thereby creating a ready‑to‑use therapeutic platform. Second, while platelet hitchhiking has been observed with synthetic peptide nanofibers [25], our HbFs achieve this mechanism using easily prepared biomaterials, with the platelet‐binding mechanism rationalized by cryo‐EM and molecular dynamics simulations. Third, this work establishes a complementary, non‐LNP, fully protein‐based platform for pulmonary mRNA delivery. Although lung‐targeted LNP systems, including SORT‐based formulations [44], also enable effective pulmonary mRNA delivery, their pulmonary cellular delivery profiles differ. For instance, 3‐Comp Lung LNPs mainly target endothelial and epithelial cells [45], and DOTAP40 Lung SORT LNPs enable delivery to basal cells [46], whereas HbFs display a fibroblast‐biased uptake pattern. More broadly, our findings extend extrahepatic mRNA delivery beyond synthetic lipids to protein‐based carriers. In addition, expanded safety studies further supported the favorable profile of mRNA@HbFs. Repeated intravenous administration for 21 days caused no detectable platelet activation (plasma sP‑selectin and PF4, Figure S20b,c), no systemic toxicity (serum TNF‑α, IL‑6, AST, ALT, BUN, and tissue histology, Figure S20a,d‐h), and no anti‑HbF humoral immunogenicity (IgM/IgG, Figure S21). In vitro, CCK‑8 assays confirmed the absence of cytotoxicity in primary MLFs, NIH/3T3, and MEF cells (Figure S22), and the formulation did not induce fibroblast activation under the tested conditions (Figure S23).

Looking ahead, the HbF platform provides a versatile foundation for the targeted pulmonary delivery of diverse nucleic acid payloads, including siRNA, CRISPR‐Cas components, and gene‐editing machinery, potentially treating a broad range of lung disorders. Further optimization of fibril formulation may enhance loading capacity and pharmacokinetics. Collectively, this research opens a promising path toward leveraging the body's innate building blocks and trafficking pathways to develop precise, effective, and biocompatible therapies for progressive lung diseases.

## Author Contributions

X.L. performed most experiments, analyzed data, prepared figures, and wrote the manuscript. S.L. determined and analyzed the atomic structure of fibrils and contributed to manuscript writing. G.W. carried out pulmonary function measurements and assisted in cellular and animal studies. S.J.L. prepared the hemoglobin fibrils. L.S.H., X.Y.L., Y.Z., W.L., and C.X.S. participated in the discussion. Q.C. supervised the structural determination and analysis. Y.F. supervised the research and provided guidance throughout the study. Y.C. conceived and designed the study, acquired funding, supervised the project, and wrote and edited the manuscript. All authors reviewed and approved the final version of the manuscript.

## Ethics Approvals

All animal procedures were approved by the Institutional Animal Care and Use Committee of Shanghai Jiao Tong University (Approval No. A2025092) and conducted in accordance with institutional guidelines.

## Conflicts of Interest

The authors declare no conflicts of interest.

## Supporting information




**Supporting File**: advs76092‐sup‐0001‐SuppMat.docx.

## Data Availability

The cryo‑EM density map and atomic coordinates of the PM1 fibril have been deposited in the Worldwide Protein Data Bank (wwPDB) and the Electron Microscopy Data Bank (EMDB) under accession codes PDB 9VE9 and EMD 64995, respectively. All other data supporting the findings are available from the corresponding author upon reasonable request.
