# Supplementary material for: Hemoglobin's α‐Helix‐to‐β‐Sheet Transition Enables Targeted mRNA Delivery to the Lung
Source: Adv Sci (Weinh). 2026 Jun 12:e76092. Online ahead of print. doi: 10.1002/advs.76092 (PMC13336868; doi:10.1002/advs.76092)
Supplement: Supplementary file 1 — Supporting File: advs76092‐sup‐0001‐SuppMat.docx. [file ADVS-9999-e76092-s001.docx]

**Supporting Information**

**Hemoglobin’s α-Helix-to-β-Sheet Transition Enables Targeted mRNA Delivery to the Lung**

*Xihua Liu^1, #^, Saiya Li^1^**^, #^, Guodong Wu^3, #^, Shuangjian Li^1^,* *Long Shuang Huang^3^, Xiaoyang Li^1^, Yiguo Zhao^1^, Wei Lu^1^, Cuixia Sun^1^, Qin Cao^2, *^, Yapeng Fang^1, 4, *^, Yiping Cao^1, *^*

^1^ Department of Food Science & Engineering, School of Agriculture & Biology, Shanghai Jiao Tong University, Shanghai 200240, China.

^2^ Bio-X Institutes, Key Laboratory for the Genetics of Developmental and Neuropsychiatric Disorders, Ministry of Education, Shanghai Jiao Tong University, Shanghai 200030, China.

^3^ Shanghai Frontiers Science Center of Drug Target Identification and Delivery, School of Pharmaceutical Sciences, Shanghai Jiao Tong University, Shanghai 200240, China.

^4^ School of Health Science & Engineering, University of Shanghai for Science and Technology, Shanghai, 200093, China.

^*^ Corresponding to: [caoyiping@sjtu.edu.cn](mailto:caoyiping@sjtu.edu.cn); [ypfang@sjtu.edu.cn](mailto:ypfang@sjtu.edu.cn); caoqin@sjtu.edu.cn

*^#^* These authors contributed equally to this work.

**Materials**

Bovine hemoglobin (Shanghai Yuanye Bio-Technology, S33492); FITC (Solarbio, F8070); Cy5.5 (MCE, HY‑D0924); Rhodamine‑wheat germ agglutinin (Rho‑WGA, MKbio, MP6326); Antifade mounting medium with DAPI (Beyotime, P0131); Red blood cell lysis buffer (Beyotime, C3702); BCA protein assay kit (Beyotime, P0009); 4-20% PAGE gels (GenScript, M42015); PVDF membranes (Millipore, IPVH00010); Dialysis membranes (3.5 kDa MWCO, Viskase; 100 kDa MWCO, Spectrum Laboratories); Glass‑bottom dishes (Cellvis, D35C4‑20‑1.5‑N); Human recombinant TGF‑β1 (PeproTech, 100‑21); Bleomycin sulfate (Yeasen Biotechnology, 60257ES25); 1.25% tribromoethanol (AibeiBio, M2910); ARCA Cy5 eGFP mRNA (ApexBio, R1009); eGFP mRNA (Novoprotein, MR010); IL‑11 scFv mRNA (custom‑synthesized by Novoprotein, sequence provided in Table S4); Tyrode’s solution (Solarbio, T1420); Poly‑L‑lysine (Shanghai Yuanye Bio-Technology, R23126); DMEM (KeyGEN BioTECH, KGL1206); Collagenase IV (Sigma‑Aldrich, C4‑28); collagenase D (Roche, 11088858001); DNase I (Yeasen Biotechnology, 10608ES25); Fetal bovine serum (Gibco, A5256701); Cell Counting Kit-8 (Beyotime, C0038); Proteinase K (Yeasen Biotechnology, 10401ES60); Universal ELISA solution kit (Beyotime, PA066S); HRP-conjugated goat anti-mouse IgG and IgM secondary antibodies (Yeasen Biotechnology, 33201ES60 and 33220ES60); Mouse platelet factor 4 (E-EL-M3080), P-selectin (E-EL-M0638), TNF-α (E-EL-M3063), and IL-6 (E-HSEL-M0003) ELISA kits were purchased from Elabscience; AST (BC1560), ALT (BC1550), and BUN (BC1535) assay kits were purchased from Solarbio. All materials were used as received unless otherwise noted.

**Preparation of apo-hemoglobin and hemoglobin fibrils (HbFs)**

Apo-hemoglobin was prepared from holo-hemoglobin using an acidified acetone method. Briefly, 6 g of bovine holo-hemoglobin was dissolved in 200 mL of ultrapure water and stirred overnight at 4 °C to ensure dissolution. 1 L of acetone containing 5 mM HCl was aliquoted into 26 centrifuge tubes and pre‑cooled at -20 °C overnight. To each tube, 2 mL of the holo-hemoglobin solution was added, followed by vortexing and centrifugation at 6000 g for 10 min at -20 °C. The resulting pellets were redissolved in ultrapure water, combined, and transferred into a dialysis membrane (MWCO 3.5 kDa). The solution was dialyzed first against ultrapure water (pH 2.0) for 12 h, and then against neutral ultrapure water with six changes. The final product was lyophilized and stored as the apo‑hemoglobin stock.

To form fibrils, the apo-hemoglobin solution (20 mg/mL in ultrapure water) was adjusted to pH 2.0 with HCl and incubated at 90 °C for 24 h. The resulting fibrils were homogenized by probe‑tip ultrasonication (Scientz‑IID, JY98; 60 W, 1s on/1s off cycle for 15 min). The sonicated fibrils were diluted in PBS to the desired concentration, adjusted to pH 7.4, and dialyzed against PBS using a membrane with 100 kDa MWCO. For in vivo studies, the HbF solution was sterilized by filtration through a 0.22 μm membrane.

**Preparation of fluorescently labeled HbFs**

A stock solution of FITC or Cy5.5 (5 mg/mL in DMSO) was added dropwise to a solution of HbFs (2 mg/mL in PBS) at a volumetric dye‑to‑protein ratio of 1:1000. The mixture was gently stirred at 4 °C for 12 h. Unreacted dye was removed by dialysis against PBS using a 3.5 kDa MWCO membrane at 4 °C. The purified FITC‑ or Cy5.5‑labeled HbFs were sterilized by filtration through a 0.22 μm membrane prior to use.

**Characterization of HbFs**

Atomic force microscopy (AFM): A 20 μL aliquot of HbF solution (0.2 mg/mL) was deposited onto a freshly cleaved mica substrate. After 2 min of adsorption, the substrate was gently rinsed with ultrapure water and dried under a gentle stream of nitrogen. Topographical images were acquired using a Bruker Multimode 8 AFM and processed by flattening with NanoScope Analysis 9.1 software.

Negative‑stain transmission electron microscopy (TEM): A 3.0 μL of HbF solution (10 mg/mL) was applied to a discharged copper grid (Beijing Zhongjingkeyi, BZ11022a). After 1 min of adsorption, excess liquid was blotted away, and the grid was rinsed three times with 3 μL ultrapure water. The sample was negatively stained with 3 μL of 2% uranyl acetate for 30 s, air-dried, and then imaged using a Talos L120C G2 TEM (Thermo Fisher Scientific) operated at 120 kV.

**Isolation of platelets, red blood cells, and white blood cells**

Platelets (PLTs): Fresh mouse peripheral blood was collected and immediately mixed with 3.2% sodium citrate. The blood was centrifuged at 500 g for 4 min at RT. The supernatant (platelet-rich plasma, PRP) was carefully collected. The PRP was then mixed with an equal volume of ACD-A buffer (39 mM citric acid, 75 mM trisodium citrate, 135 mM glucose, pH 6.5-6.8) and centrifuged at 1250 g for 8 min at RT. The platelet pellet was gently resuspended in ACD-A buffer and washed by centrifugation at 800 g for 6 min. Purified platelets were finally resuspended in HEPES‑Tyrode’s buffer[1, 2].

Red blood cells (RBCs): The anticoagulated whole blood was centrifuged at 500 g for 4 min at RT. The supernatant plasma and the intermediate buffy coat (containing white blood cells) were carefully removed. The RBC pellet was resuspended in a 10-fold volume of PBS containing 0.5% BSA, gently mixed, and centrifuged at 500 g for 5 min at RT. This washing step was repeated twice. Purified RBCs were finally resuspended in HEPES-Tyrode’s buffer^1^.

White blood cells (WBCs): The anticoagulated whole blood was treated with a ten‑fold volume of red blood cell lysis buffer. The mixture was gently inverted for 90 s until the color changed from red to clear, indicating complete lysis of RBCs. An equal volume of PBS was then added to stop the lysis. The sample was centrifuged at 300 g for 5 min. The supernatant was discarded, and the resulting white cell pellet, comprising the WBCs, was collected. Purified WBCs were resuspended in HEPES-Tyrode’s buffer[1, 2].

**Identification of HbF binding preference to blood cell types**

CLSM: Glass-bottom dishes were coated with 0.1 mg/mL poly‑L‑lysine at 37 °C for 20 min, followed by gentle washing. To visualize platelets, platelet suspension was pre‑labeled with 5 µg/mL Rho‑WGA in the dark at RT for 10 min. The labeled platelets were then mixed with RBCs or WBCs. The mixed cell suspension was added to the pretreated dish and allowed to settle for 5 min at 37 °C for mild attachment. After carefully removing the supernatant, pre‑warmed FITC‑HbF solution (2 mg/mL) was added and incubated for 1 min at 37 °C. The solution was aspirated, and cells were fixed with 4% paraformaldehyde for 10 min at RT. After washing with PBS, samples were mounted with an antifade mounting medium containing DAPI. Images were acquired using a Leica STELLARIS 5 confocal microscope.

Flow cytometry: Isolated platelets, RBCs, and WBCs were pooled and resuspended in HEPES‑Tyrode’s buffer. The cell suspension was incubated with pre‑warmed FITC‑HbF (2 mg/mL) at 37 °C for 1 min with gentle agitation. Cells were then centrifuged at 300 g for 5 min, washed once with PBS containing 0.5% BSA and 2 mM EDTA, and resuspended in HEPES‑Tyrode’s buffer. Subsequently, cells were stained at 4 °C for 30 min with the following antibodies: PE anti‑CD41 (BioLegend, 133905), APC anti‑TER‑119 (BioLegend, 116211), and Brilliant Violet 421 anti‑CD45 (BioLegend, 103133). Samples were analyzed on a Cytek® Aurora full‑spectrum flow cytometer to quantify HbF binding to each cell population.

**Cryo-EM data collection and processing**

HbF sample was applied to glow-discharged Quantifoil R1.2/1.3 200-mesh Cu grids. Vitrification was performed using a Vitrobot Mark IV (Thermo Fisher Scientific) with 3.0 µL of sample, 3 s blot time, and plunge-freezing in liquid ethane. Cryo‑EM data were collected on a Titan Krios transmission electron microscope (Thermo Fisher Scientific) operating at 300 kV, equipped with a K3 direct electron detector. Micrographs were recorded at a nominal magnification of 130,000×, corresponding to a physical pixel size of 0.932 Å, with a total accumulated dose of 40 e⁻ Å⁻².

All data processing was performed within the RELION 4.0[3] framework unless otherwise stated. A total of 2,430 micrographs were subjected to beam‑induced motion correction using MotionCor2[4] and contrast transfer function (CTF) estimation with CTFFIND‑4.1.14[5]. Fibril particles were automatically picked using Topaz v0.2.5[6] and extracted at two box sizes (1024 and 360 pixels) for subsequent analysis. Reference-free 2D classification of the 1024‑pixel particles revealed four distinct structural populations, designated PM1 (85.6%), PM2 (3.7%), PM3 (7.6%), and PM4 (3.1%). Particles belonging to the dominant PM1 class (360‑pixel box) were selected for high-resolution reconstruction. Initial helical parameters, determined from 2D averages, were refined to a rise of 4.782 Å and a twist of -1.591°. Multiple rounds of 3D classification were conducted to isolate a homogeneous subset of particles, which were then subjected to gold‑standard refinement, resulting in a near‑atomic‑resolution map.

**Atomic model building and refinement**

A sharpened cryo-EM map of the PM1 at 3.3 Å resolution was obtained using phenix.sharpen[7]. De novo model building was performed with ModelAngelo 1.0[8], using the sequences of bovine hemoglobin subunit alpha (Uniprot ID: P01966) and subunit beta (Uniprot ID: P02070) as input. The density was unambiguously assigned to the hemoglobin subunit beta sequence. Manual adjustments for optimal fit were made in COOT v.0.9.8.1[9]. A five‑layer fibril model was constructed in silico and refined against the map using phenix.real_space_refine (Phenix v.1.21.1). Model quality was validated with MolProbity[10], yielding favorable statistics for clashes, Ramachandran outliers, and overall geometry (Table S1).

**Molecular dynamics simulations**

A computational model of the platelet membrane bilayer was constructed using CHARMM-GUI[11]. The asymmetric bilayer was composed of an upper leaflet containing 36 cholesterol, 54 POPC, 6 POPS, and 24 PSM molecules, and a lower leaflet containing 24 cholesterol, 24 POPC, 36 POPE, 24 POPS, and 12 POPI molecules (Table S2). The bilayer was solvated with a TIP3P water layer extending at least 2.0 nm on each side. Any water molecules penetrating the hydrophobic core of the bilayer were removed using VMD[12]. The system was neutralized with 65 Na⁺ and 23 Cl⁻ ions. Initial equilibration of the lipid bilayer was performed by running a 14.7 ns molecular dynamics (MD) simulation in the NPT ensemble using the CHARMM36m force field[13], until the membrane area stabilized (Fig. S21). The resulting equilibrated membrane patch was used for all subsequent simulations.

A ten-chain structural model of HbF was generated using a custom Python script[14]. This fibril model was then positioned above the pre-equilibrated membrane surface at an appropriate distance using VMD. Four independent simulation systems were prepared, differing in the initial rotational orientation of the fibril relative to the membrane (detailed in Table S3). For each system, the simulation box was extended and solvated. Water molecules overlapping with the coordinates of either the lipid bilayer or the fibril were deleted. Each system was then neutralized by adding 22 Na⁺ ions.

All MD simulations were performed using GROMACS. Each system first underwent a 200 ps NPT equilibration with a 1 fs timestep, applying positional restraints on the protein and lipid heavy atoms. This was followed by a 100 ns production simulation in the NPT ensemble with a 2 fs timestep and no restraints. Simulations were conducted at 310 K, maintained using the V-rescale thermostat. Semi-isotropic pressure coupling was applied at 1 bar using the Parrinello-Rahman barostat. This setup allowed for the observation of the spontaneous binding interaction between the HbF and the membrane surface. The solvent-accessible surface area (SASA) at the HbF-membrane interface was calculated from the simulation trajectories using the gmx sasa tool within the GROMACS package.

**Isolation and culture of primary mouse lung fibroblasts (MLFs)**

Primary MLFs were isolated from euthanized C57BL/6 mice. Lung tissues were dissected, minced, and digested in 1 mg/mL collagenase IV at 37 °C for 30 min. Digestion was terminated by adding three volumes of DMEM. The mixture was centrifuged at 1700 rpm for 5 min, and both the tissue layer and cell pellet were collected. After two washes with DMEM, the final pellet was resuspended in DMEM supplemented with 10% fetal bovine serum (FBS) and seeded into a 10 cm culture dish. Cells were maintained at 37 °C in a humidified 5% CO₂ incubator (Thermo Scientific, Heracell 150i). After 4-5 days, when cells reached confluence, they were filtered through a 45 μm cell strainer to obtain a purified fibroblast population for subsequent experiments. The transition from fibroblast to myofibroblast is induced by the addition of 10 ng/mL TGF‑β1.

**Formation and characterization of mRNA@HbF Complexes**

Complexes were formed via electrostatic self‑assembly at room temperature. HbF solution (2 mg/mL in PBS, pH 7.4) was mixed with mRNA at a mass ratio of 5:1 (HbF:mRNA). The mixture was gently agitated for 30 min and then dialyzed (MWCO 100 kDa) against sterile PBS at 4 °C for 12 h to remove unbound mRNA.

The zeta potential of the complexes was measured using a Zetasizer Nano‑ZS90 (Malvern Instruments). Morphology was assessed by TEM as previously described. Intracellular co‑localization of FITC‑ HbF and Cy5‑mRNA was visualized by confocal microscopy.

**Cellular uptake**

MLFs were seeded in 6‑well plates or glass‑bottom dishes at a density of 10⁵ cells per well. Cells were treated with 0.5 µg of eGFP mRNA@HbF. As a positive control, an equivalent amount of free eGFP mRNA was transfected using Lipofectamine 2000 according to the manufacturer’s protocol. After 24 h, eGFP expression was visualized using a Leica STELLARIS 5 confocal microscope. For quantification, cells were harvested, washed with PBS, and analyzed by flow cytometry (BD Fortessa X20) to determine the percentage of eGFP^+^ cells.

The same procedure was applied to mouse embryonic fibroblasts (MEFs, Shang Fuheng Biotechnology) and NIH/3T3 cells (Cell Bank of the Chinese Academy of Sciences). Fluorescence intensity was quantified using ImageJ software.

**Lysosomal escape analysis**

The endolysosomal escape of mRNA@HbF complexes was monitored in MLFs using complementary markers for lysosomes and late endosomes. Cells were incubated with complexes containing Cy5-labeled eGFP mRNA for specified durations (0, 1, 3, 5 h). For live-cell tracking, cells were stained with LysoTracker Green (50 nM, 30 min) and Hoechst 33342, then imaged in live-cell imaging solution. For fixed-cell analysis, cells were stained with an anti-Rab7 primary antibody (1:200) followed by a fluorescent secondary antibody, with nuclei counterstained by DAPI. All images were acquired on a Leica STELLARIS 5 confocal microscope. Colocalization between the Cy5-mRNA signal (red) and organelle markers (green) was quantified by calculating the Pearson correlation coefficient (*r*) using ImageJ, with a progressive decrease in *r* indicating efficient endosomal escape.

**Migration assay**

Cell migration was assessed using Transwell® inserts (8.0 μm pore, Corning). MLFs were seeded into the upper chamber and allowed to adhere overnight. Cells were then treated for 24 h with PBS, free IL‑11 scFv mRNA, or IL‑11 scFv mRNA@HbF. After treatment and a subsequent 24‑h serum‑starvation period, TGF‑β1 (10 ng/mL) was added to the lower chamber as a chemoattractant. Following a 24‑h incubation, non‑migratory cells on the upper membrane surface were removed with a cotton swab. Migrated cells on the lower surface were fixed with 4% paraformaldehyde, stained with 0.1% crystal violet, and imaged using an Olympus microscope. Migrated cells were counted using ImageJ software.

**Wound‑healing assay**

MLFs were seeded and allowed to form a confluent monolayer. A uniform scratch was created using a sterile pipette tip. Cells were then treated with PBS, free IL‑11 scFv mRNA, or IL‑11 scFv mRNA@HbF in the presence of TGF‑β1 (10 ng/mL) for 24 h. The initial wound area (0 h) and the area after 24 h of incubation in serum‑free medium were imaged. The percentage of wound closure was quantified using ImageJ.

**Animal studies**

All animal procedures were approved by the Institutional Animal Care and Use Committee of Shanghai Jiao Tong University (Approval No. A2025092) and conducted in accordance with institutional guidelines. Male BALB/c mice (6-8 weeks old) were housed under specific‑pathogen‑free conditions.

Pulmonary fibrosis was induced by a single intratracheal administration of bleomycin sulfate (2 U/kg). Mice were randomly assigned to treatment groups and received intravenous injections every 3 days from day 7 to day 21 post‑bleomycin. Treatments included PBS, free IL‑11 scFv mRNA (15 µg/mouse), and IL‑11 scFv mRNA@HbF at low‑dose (10 µg mRNA/mouse) and high‑dose (15 µg mRNA/mouse) levels. Body weight was monitored every 2 days.

**In vivo and ex vivo fluorescence imaging**

Mice were administered Cy5.5‑HbF or PBS via tail vein injection. Real‑time biodistribution was monitored at designated time points using an IVIS Spectrum Imaging System (PerkinElmer; Ex/Em = 680/710 nm). After in vivo imaging, mice were euthanized, and major organs were harvested for ex vivo fluorescence imaging.

**Biodistribution in lung cell subpopulations**

Mice were intravenously administered Cy5.5‑HbF (2 mg/mL, 200 μL per mouse) or an equal volume of PBS. After 6 h, lungs were harvested, minced, and digested in HBSS containing 1 mg/mL collagenase D and 0.1 mg/mL DNase I at 37 °C for 1 h with gentle agitation. Digestion was quenched by adding an equal volume of complete DMEM medium. The cell suspension was filtered through a 40 μm cell strainer, washed once by centrifugation, and treated with red blood cell lysis buffer. After washing, cells were stained with a viability dye (Thermo Fisher, 88‑8824‑00) for 30 min at room temperature in the dark. Cells were then blocked in PBS containing 5% FBS for 30 min and incubated for 30 min at 4 °C with the following fluorescently conjugated antibodies: Spark PLUS UV395‑anti‑CD45 (BioLegend, 103192), FITC‑anti‑CD326 (BioLegend, 118207), Brilliant Violet 421‑anti‑CD31 (BioLegend, 102423), and PE‑anti‑CD140α (BioLegend, 135905). The uptake of Cy5.5‑HbF in distinct lung cell populations was analyzed by flow cytometry (BD LSRFortessa X20).

**Immunohistochemistry**

Paraffin‑embedded lung sections (5 µm) were deparaffinized, rehydrated, and subjected to antigen retrieval in Tris/EDTA buffer (pH 9.0) via a heated water bath. Endogenous peroxidase activity was blocked with 3% H₂O₂. Sections were incubated overnight at 4 °C with primary antibodies: anti‑IgG (Proteintech, 98136‑1RR, 1:500) or anti‑GFP (Proteintech, 50430‑2‑AP, 1:100). After washing, appropriate HRP‑conjugated secondary antibodies were applied. Staining was developed with 3,3′‑diaminobenzidine (DAB; ZSGB‑BIO, ZLI‑9018), and sections were lightly counterstained with hematoxylin, dehydrated, and mounted. Whole‑slide images were acquired using a 3DHISTECH digital slide scanner.

**Immunofluorescence staining**

For cellular staining: MLFs grown on glass‑bottom dishes were fixed with 4% paraformaldehyde, permeabilized with 0.2% Triton X‑100, and blocked with 3% BSA. Cells were incubated overnight at 4 °C with primary antibodies against ACTA2 and COL1A1 (both 1:200), followed by incubation with a goat anti‑rabbit IgG (H+L) secondary antibody (BOSTER, BA1032, 1:300).

For tissue staining: Paraffin‑embedded lung sections (5 μm) were deparaffinized, rehydrated, and subjected to antigen retrieval. The staining procedure followed the cellular IF protocol, using the following primary antibodies: anti‑CD326 (Proteintech, 21050‑1‑AP, 1:500), anti‑CD31 (Abcam, ab222783, 1:200), anti‑CD45 (HUABIO, HA723506, 1:200), anti‑CD140α (Thermo Fisher, 14‑1401‑81, 1:200), anti‑ACTA2 (CST, 48938, 1:200), anti‑COL1A1 (CST, 72026, 1:200), and anti‑FN1 (CST, 63779, 1:200).

For eGFP autofluorescence imaging: Deparaffinized and rehydrated lung sections were counterstained with DAPI and imaged directly in the GFP channel.

**Western blotting**

Cells or lung tissues were lysed in RIPA buffer supplemented with 1 % protease inhibitor cocktail. Protein concentration was determined by BCA assay. Equal amounts of protein were separated by 4-20% gradient SDS‑PAGE gels, transferred to PVDF membranes, and blocked. Membranes were incubated overnight at 4 °C with primary antibodies: anti-GAPDH (Abcam ab181602, 1:10,000), anti-ACTA2 (CST 48938, 1:1000), anti-COL1A1 (CST 72026, 1:1000), anti-FN1 (CST 63779, 1:1000), anti-IL11 (Proteintech 55169-1-AP, 1:1000), anti-MMP2 (Affinity AF0577, 1:1000), anti-Smad2/3 (CST 8685T, 1:1000), anti-P-Smad2 (CST 3108S, 1:1000), anti-P-Smad3 (CST 9520S, 1:1000). After incubation with horseradish peroxidase-conjugated secondary antibodies at room temperature, protein signals were visualized using the Tanon Imaging System. Band intensities were quantified using ImageJ software.

**Hydroxyproline assay**

Lung collagen content was quantified by measuring hydroxyproline levels using a commercial colorimetric assay kit (Elabscience, E-BC-K062-M) according to the manufacturer’s protocol. Briefly, ~100 mg of lung tissue was hydrolyzed in acid at 95 °C for 6 h. The hydrolysate was neutralized, reacted with assay reagents, and absorbance was measured at 558 nm using a FLUOstar Omega microplate reader.

**Histopathological analysis**

Paraffin‑embedded lung sections (5 µm) were stained with hematoxylin and eosin (H&E), Masson’s trichrome, or Sirius Red. Whole‑slide digital images were acquired using an SQS‑120P slide scanner. Fibrosis severity was scored blindly by two independent observers using the Ashcroft scale (0–8)[15] on H&E‑stained sections. The Ashcroft criteria are as follows: 0, normal lung structure; 1, minimal fibrous thickening of alveolar/bronchiolar walls; 2–3, moderate thickening without architectural distortion; 4–5, increased fibrosis with definite structural damage and fibrous bands; 6–7, severe distortion and large fibrous areas; 8, total fibrous obliteration.

Collagen deposition was quantified on Masson’s trichrome‑stained sections (blue area) and Sirius Red‑stained sections (positive red area) using ImageJ software, expressed as the percentage of stained area relative to the total tissue area.

**Pulmonary function test**

On day 21, mice were deeply anesthetized and tracheostomized with a 14‑gauge catheter. Pulmonary function was assessed using the FlexiVent system (SCIREQ) under resting ventilation. Respiratory system resistance (Rrs), elastance (Ers), and compliance (Crs) were measured by the forced oscillation technique. Inspiratory capacity (IC) was determined via a deep inflation maneuver. Forced vital capacity (FVC) and forced expiratory volume at 0.2 s (FEV0.2) were obtained using a negative pressure‑forced expiration maneuver. Static compliance (Cst) was derived from the pressure–volume (PV) curve.

**Anti-HbF IgM/IgG indirect ELISA**

Serum samples collected at day 0 and day 21 were analyzed by indirect ELISA using HbF- or BSA-coated 96-well plates (5 μg/mL, overnight, 4 °C). BSA-coated wells were included as a non-specific binding/background control. After washing and blocking, serum samples were incubated for 1 h, followed by HRP-conjugated goat anti-mouse IgM or IgG secondary antibody for 1 h. Plates were developed with TMB for 10 min, stopped, and read at 450 nm. HbF-specific signal was calculated as $HbF specific signal=\left( {OD}_{HbF}-{OD}_{blank} \right)-\left( {OD}_{BSA}-{OD}_{blank} \right)$, where blank wells contained sample dilution buffer instead of serum.

**Statistical analysis**

Data are presented as mean ± SEM. Where applicable, quantification of protein expression and fluorescence intensity was normalized to the corresponding control group before statistical analysis. Sample sizes are indicated in the corresponding figure legends. Statistical analysis was performed using GraphPad Prism 9.0. For comparisons between two groups, an unpaired two-tailed Student’s t-test was used; for comparisons among three or more groups, one-way ANOVA followed by Tukey’s multiple comparisons test was used. A p‑value < 0.05 was considered statistically significant. Graphic was created by the authors using BioRender (https://app.biorender.com) under license.


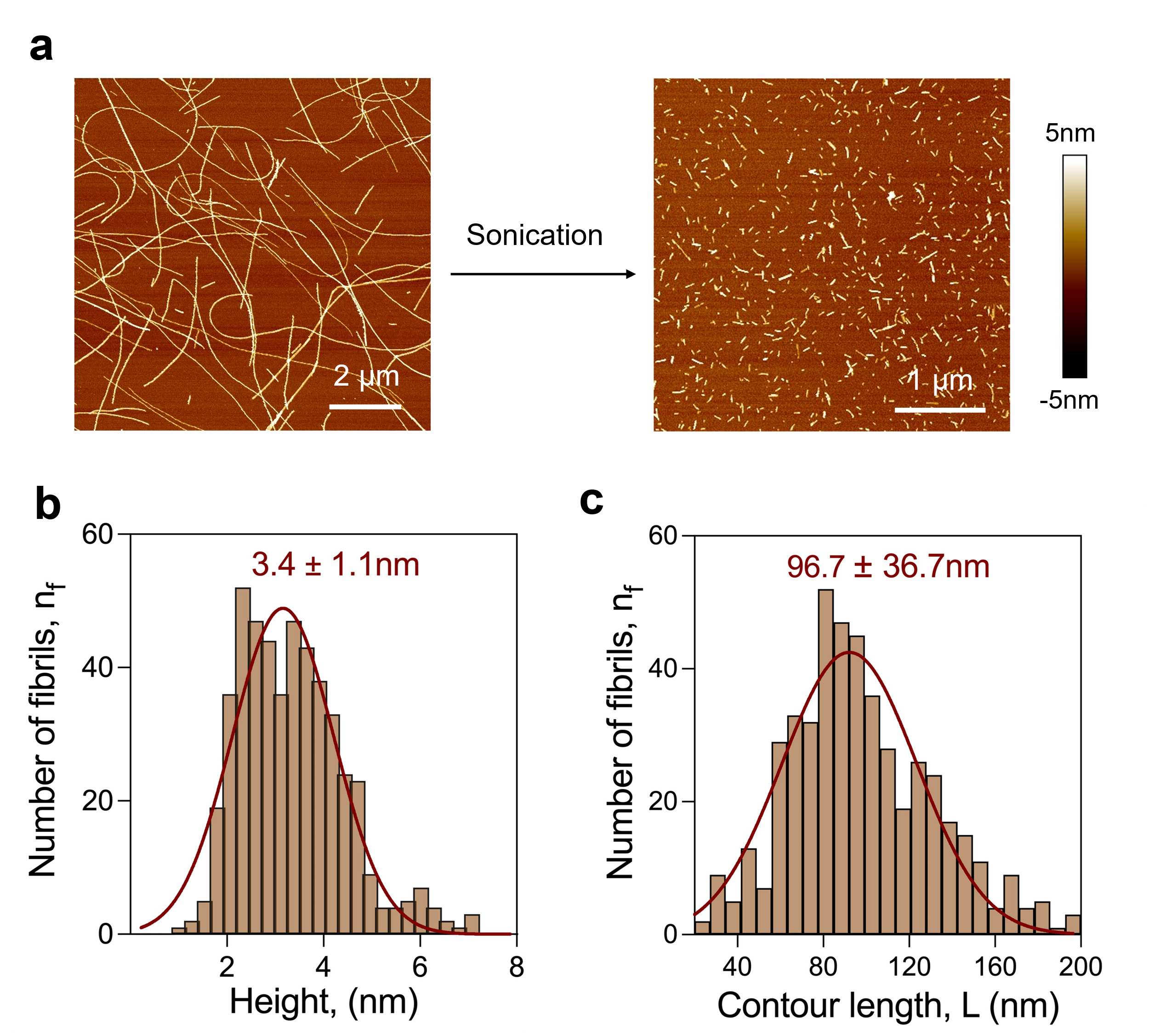


**Figure S1. Characterization of hemoglobin fibrils (HbFs).** (a) AFM images of HbFs before and after sonication. (b) Height distribution and (c) contour length distribution of the sonicated HbFs.


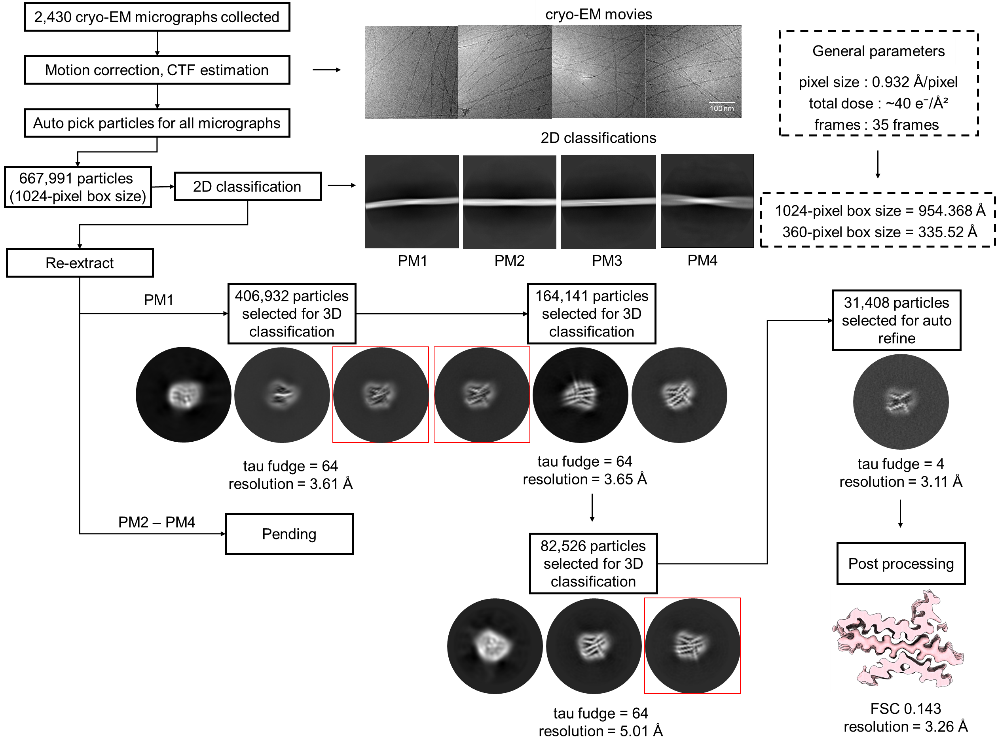


**Figure S2. Workflow of cryo-EM data processing for HbF structure determination.**


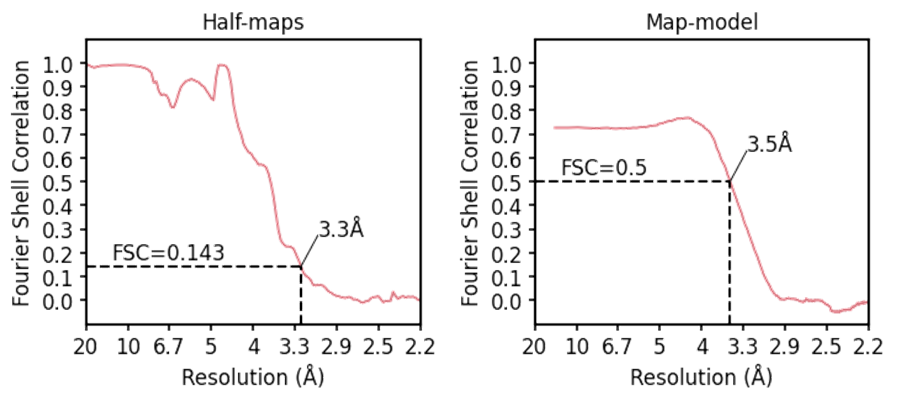


**Figure S3. Resolution assessment of the PM1 reconstruction.** (Left panel) Fourier shell correlation (FSC) curve between two independently refined half-maps, indicating a global resolution of 3.3 Å at 0.143 cutoff. (Right panel) FSC curve between the final atomic model and the reconstructed map, showing a resolution of 3.5 Å at 0.5 cutoff.


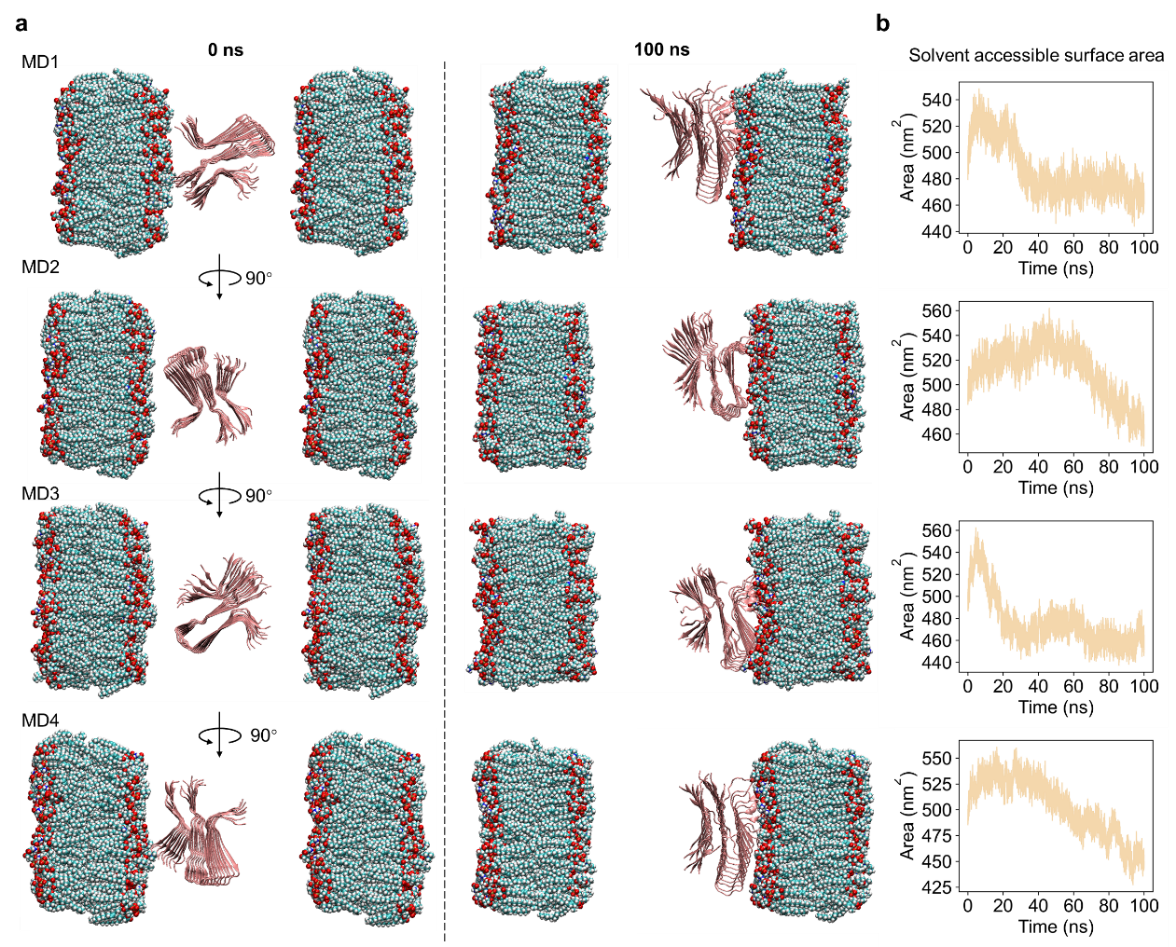


**Figure S4. MD simulations of HbF binding to platelet membrane.** (a) Overview of the four independent simulation systems, showcasing the different initial orientations of HbF relative to the membrane prior to simulation. All systems converged to a similar binding mode after 100 ns. (b) Time-dependent change in the solvent accessible surface area (SASA) at the fibril-membrane interface throughout the 100 ns run.

**
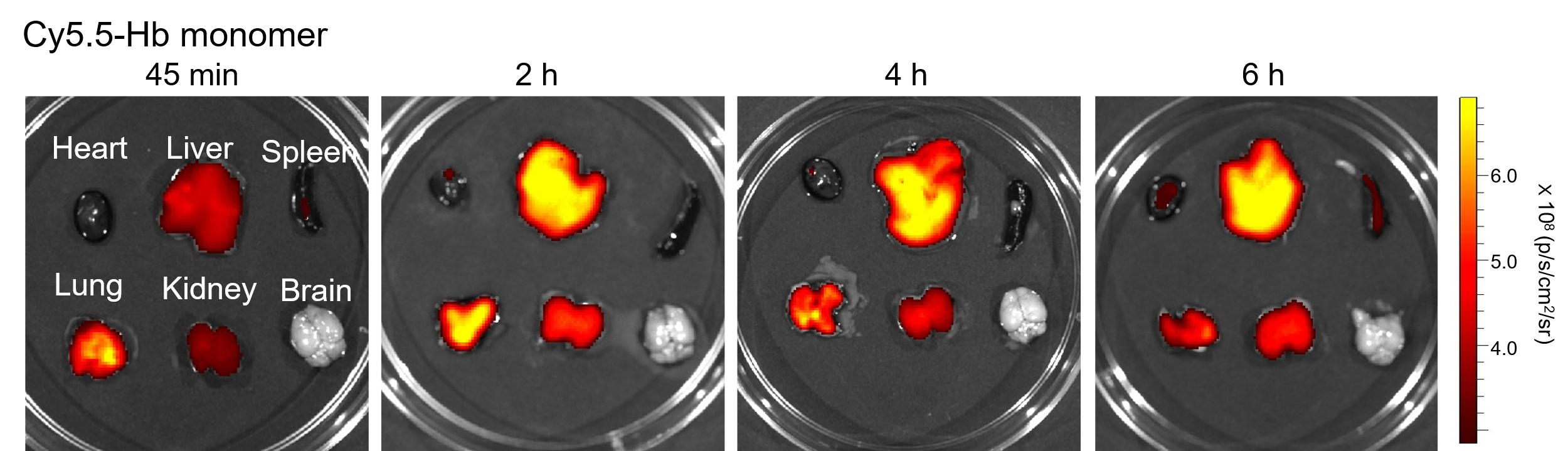
**

**Figure S5. Ex vivo fluorescence imaging of major organs.** Images show organs harvested at indicated time points after intravenous injection of Cy5.5‑labeled native Hb (2 mg/mL, 200 µL/mouse).

**
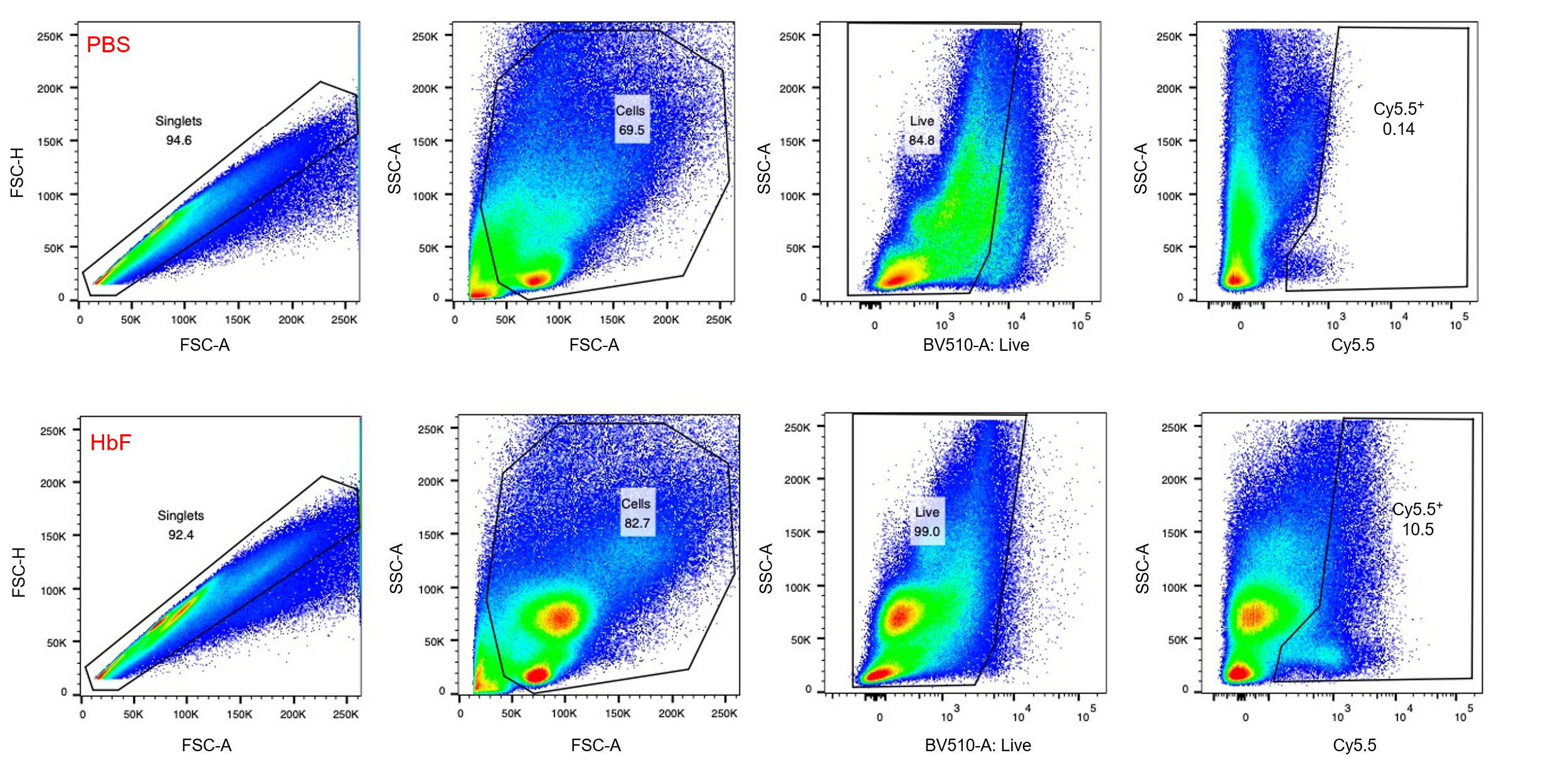
**

**Figure S6.** The gating strategy of Figure 4c.


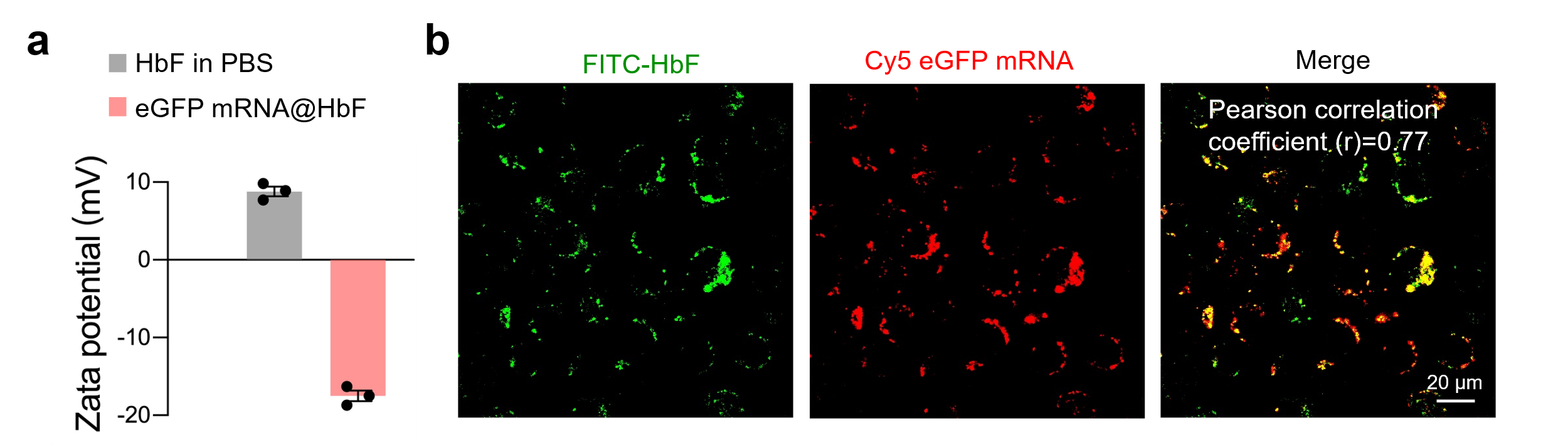


**Figure S7. Characterization of eGFP mRNA@HbF.** (a) Zeta potential of HbFs and eGFP mRNA@HbF. (b) Colocalization analysis of FITC‑HbF (green) and Cy5 eGFP mRNA (red) in NIH/3T3 cells. The Pearson correlation coefficient is 0.77.

**
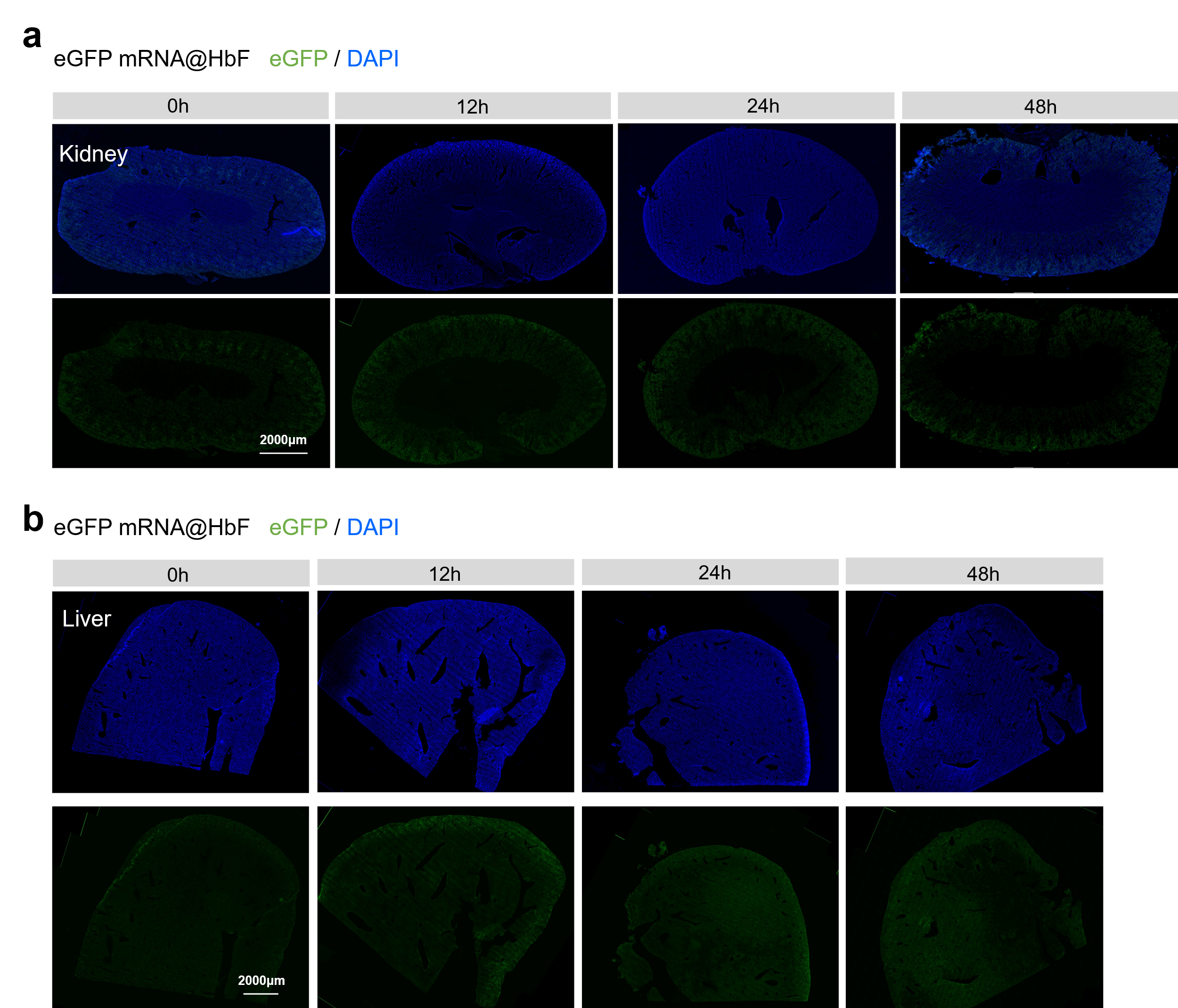
**

**Figure S8. Representative images showing absence of eGFP fluorescence in kidney and liver after eGFP mRNA@HbF administration.** Tissue sections from (a) kidney and (b) liver were collected at the indicated time points after intravenous injection of eGFP mRNA@HbF (10 μg mRNA/mouse). Green: eGFP signal; Blue: DAPI (nuclei).


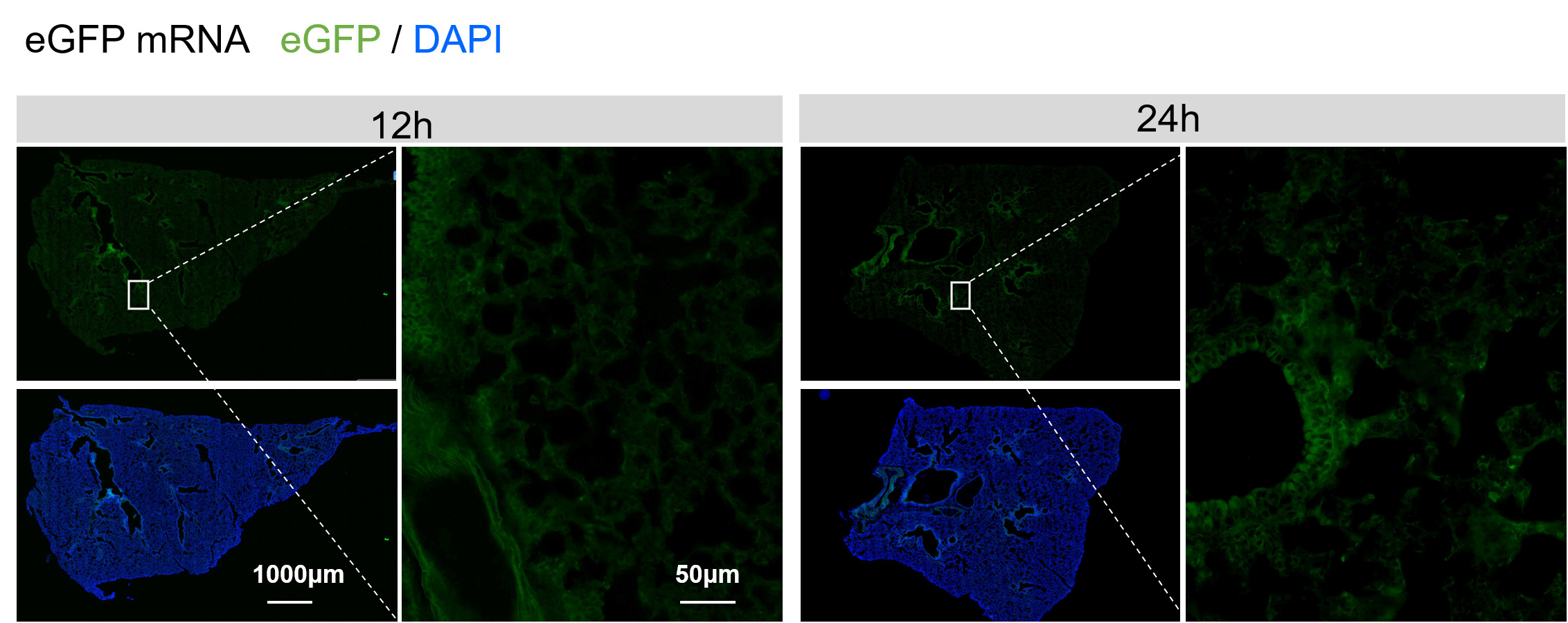


**Figure S9. Free eGFP mRNA fails to induce detectable expression in lung tissue.** Lung sections were collected at 12 h and 24 h after intravenous injection of free eGFP mRNA (10 μg mRNA per mouse). Green: eGFP signal; Blue: DAPI (nuclei).

**
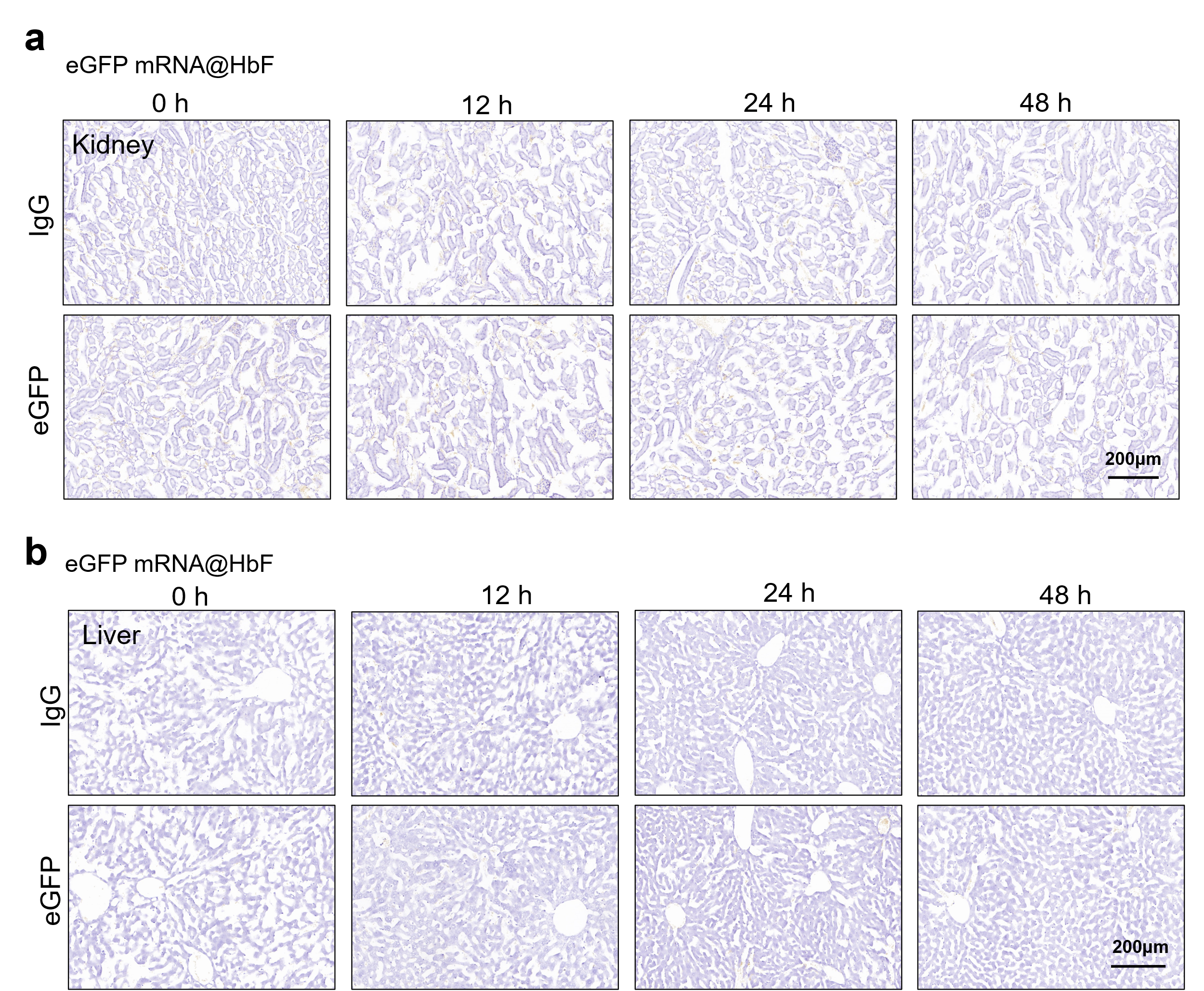
**

**Figure S10. Representative images showing absence of eGFP protein in kidney and liver by immunohistochemistry after eGFP mRNA@HbF administration.** Immunohistochemical staining of (a) kidney and (b) liver sections at the indicated time points post-injection. An IgG isotype control was used to confirm staining specificity.


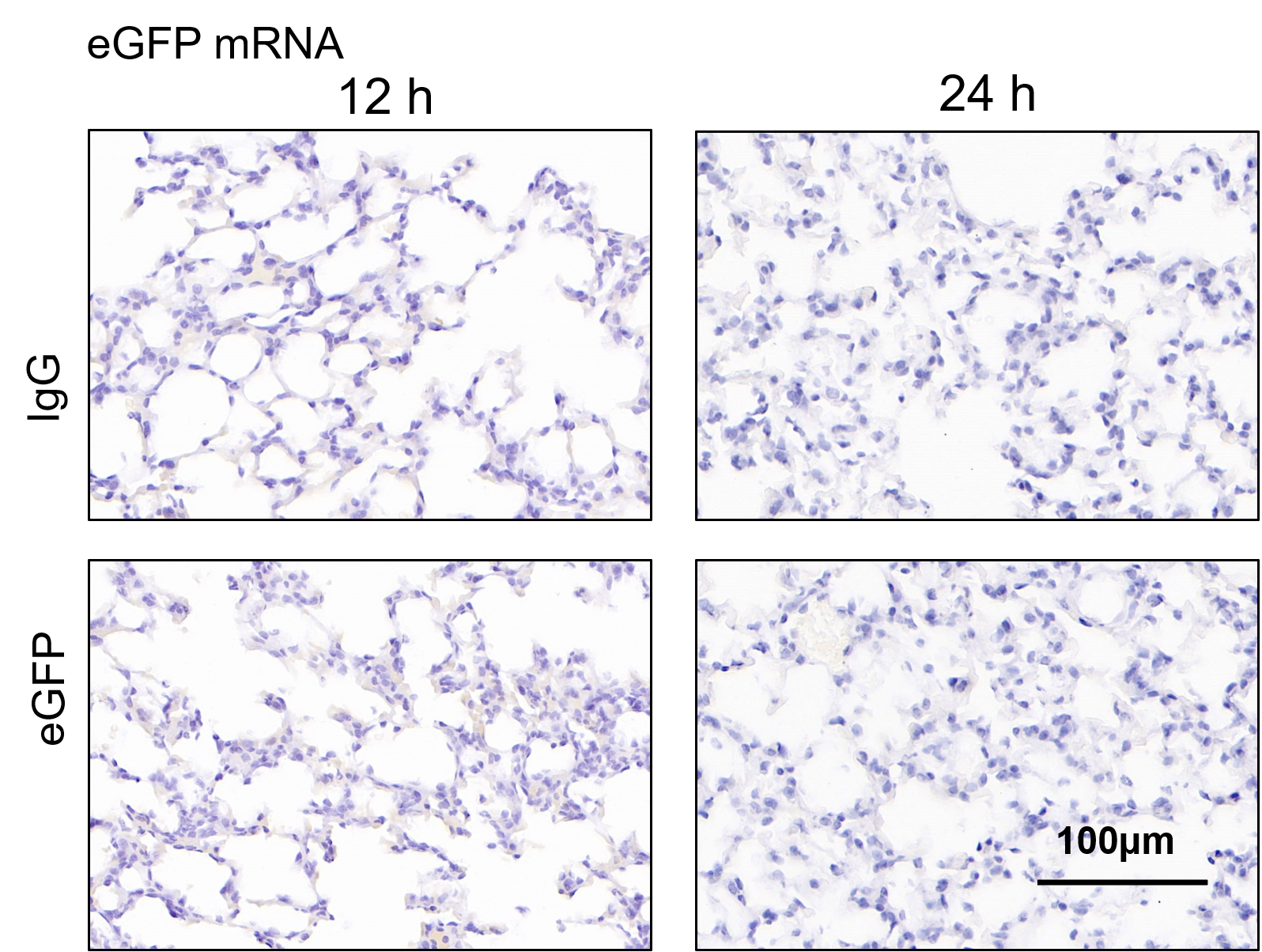


**Figure S11. Immunohistochemistry confirms absence of eGFP protein in lung after free mRNA administration.** Immunohistochemical staining of lung sections at 12 h and 24 h after intravenous injection of free eGFP mRNA (10 μg mRNA per mouse). An IgG isotype control was included.


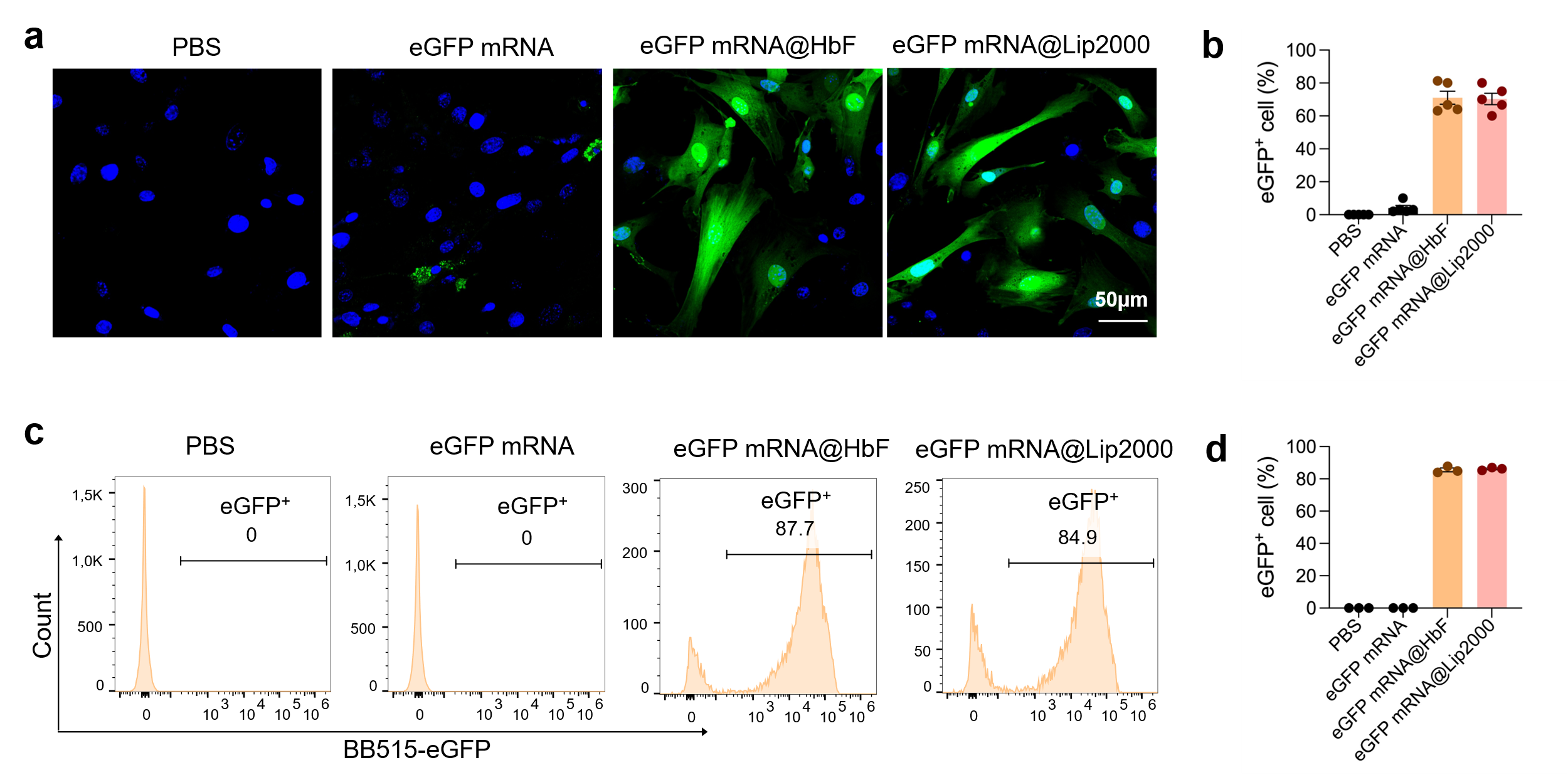


**Figure S12.** **eGFP mRNA@HbF enables efficient eGFP expression in primary MLFs.** (a) Representative immunofluorescence images and (b) quantification of eGFP expression in primary MLFs after 24 h incubation with PBS, free eGFP mRNA, eGFP mRNA@HbF (0.5 μg/mL), or eGFP mRNA@Lip2000 (0.5 μg/mL, positive control). (c) Representative flow cytometry histograms and (d) quantification of eGFP⁺ MLFs under the same treatment conditions; Data are presented as mean ± SEM (n≥3).


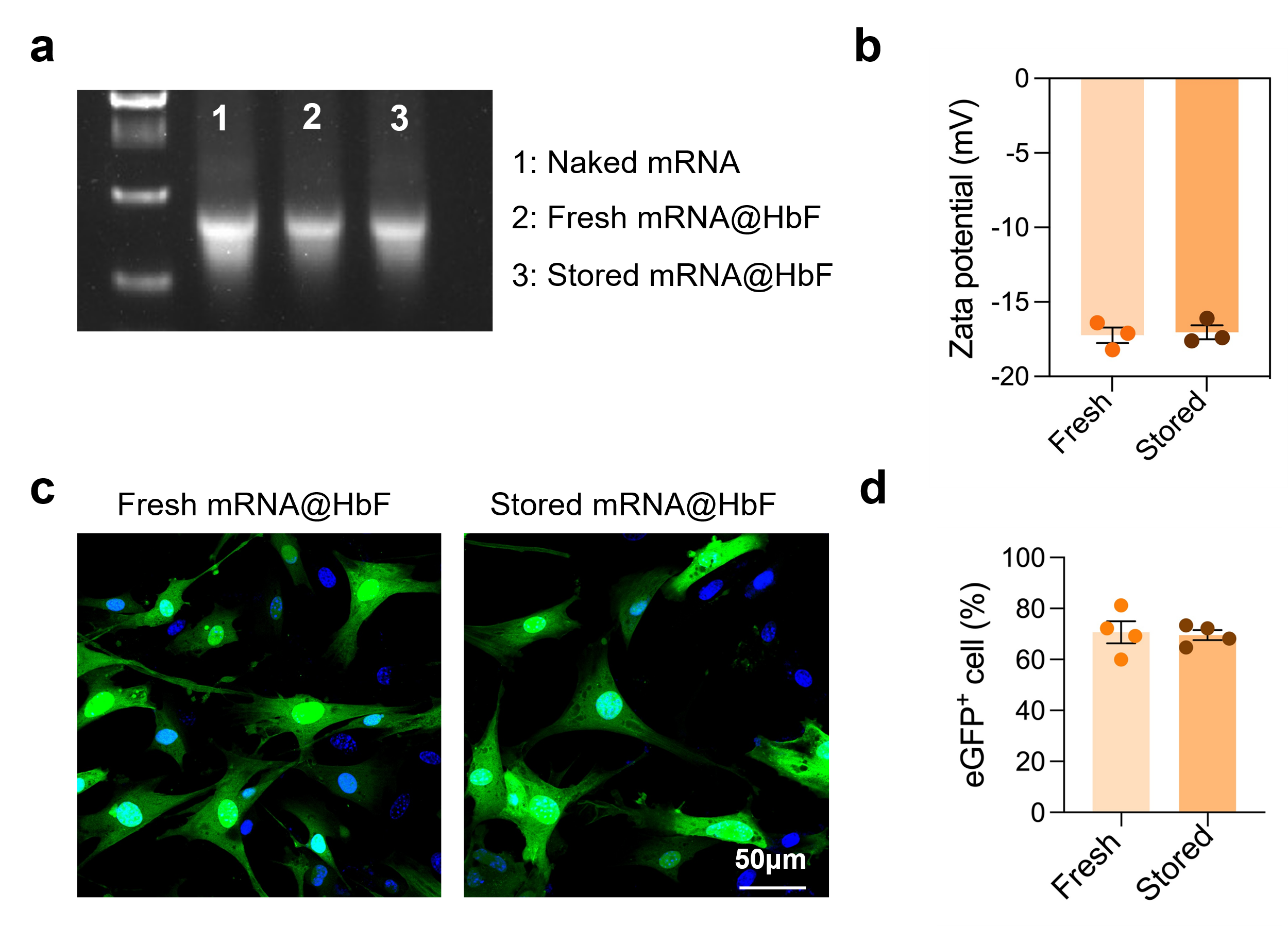


**Figure S13. One month storage stability of eGFP mRNA@HbFs.** (a) Agarose gel electrophoresis of naked mRNA (lane 1), fresh mRNA@HbF (lane 2), and 1-month stored mRNA@HbF (lane 3). For lanes 2 and 3, mRNA was released from HbF complexes by proteinase K treatment (final concentration, 0.5 mg/mL) before electrophoresis. (b) Zeta potential of fresh and stored mRNA@HbFs, n=3. (c) Representative fluorescence images of MLFs treated with fresh or stored mRNA@HbFs (0.5 μg/mL) and (d) Quantification of eGFP⁺ cells, n=4. Stored eGFP mRNA@HbFs were kept at 4 °C in PBS for 1 month before use. Data are presented as mean ± SEM.


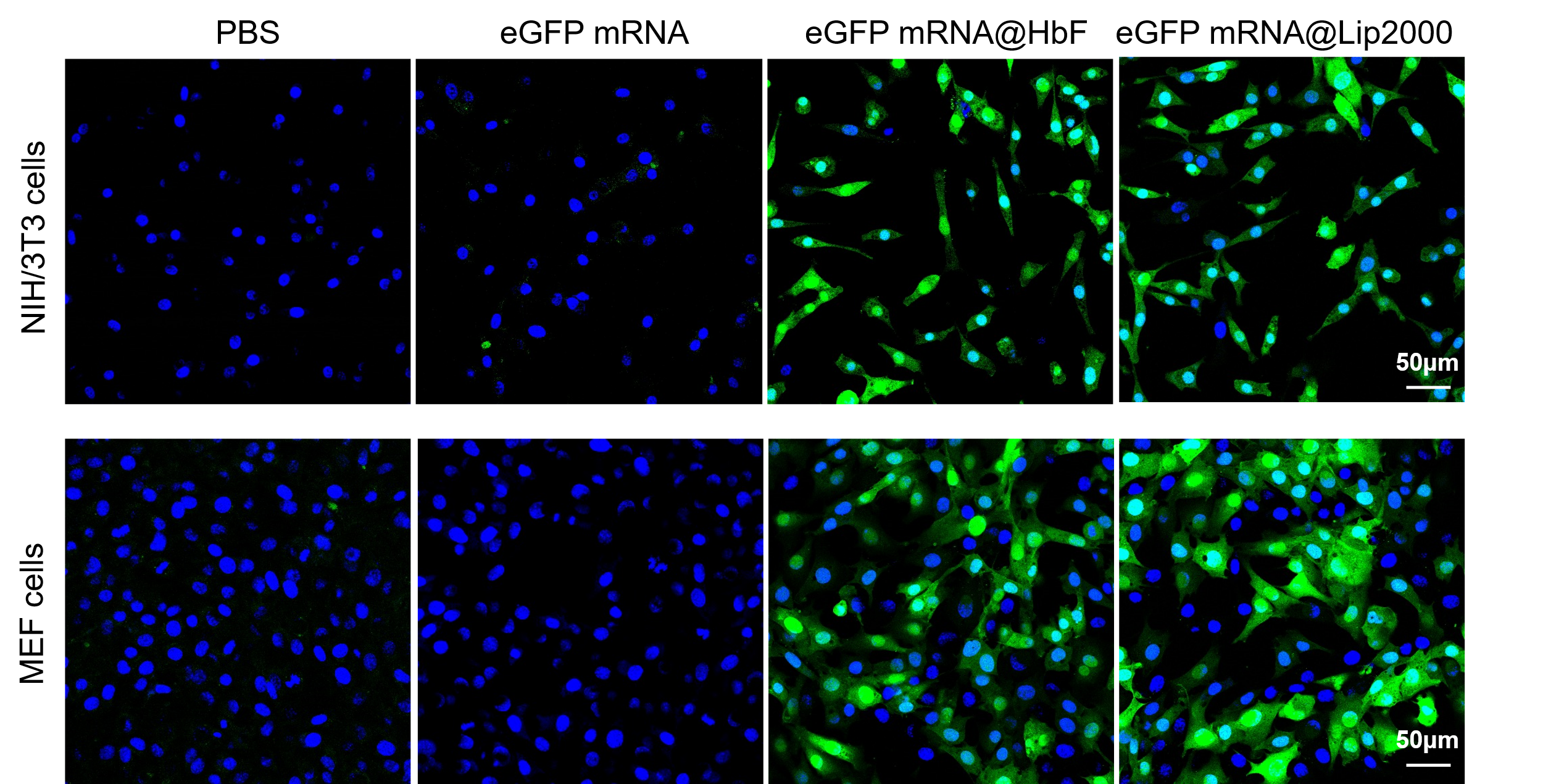


**Figure S14.** **eGFP mRNA@HbF enables efficient eGFP expression in NIH/3T3 and MEF cell lines.** Representative immunofluorescence images of NIH/3T3 (top) and MEFs (bottom) after 24 h treatment with PBS, free eGFP mRNA, eGFP mRNA@HbF (0.5 μg/mL), or eGFP mRNA@Lip2000 (0.5 μg/mL, positive control).


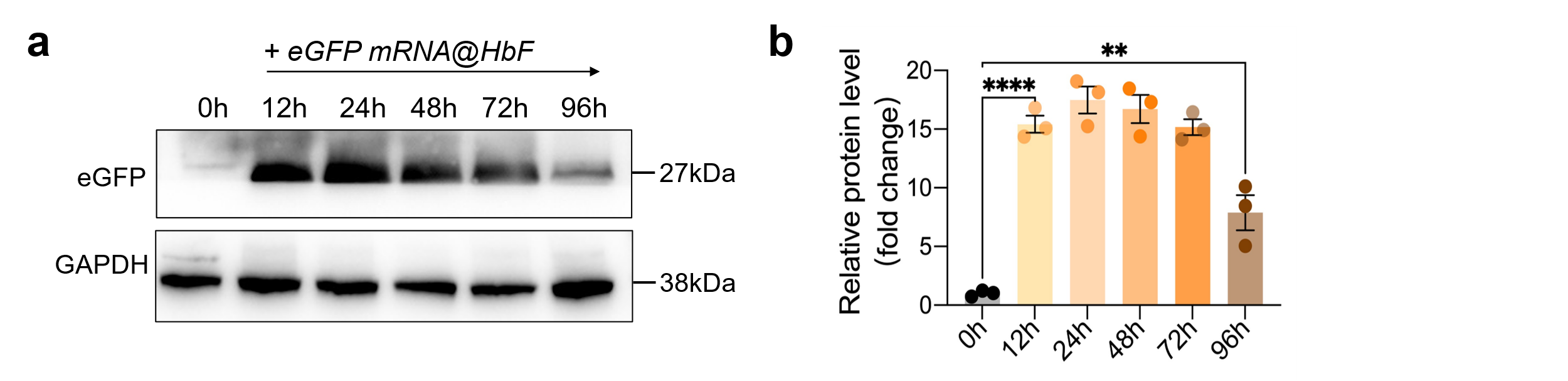


**Figure S15.** **Sustained eGFP expression in MLFs after eGFP mRNA@HbF treatment.** (a) Representative Western blots and (e) quantification of eGFP expression in MLFs at indicated time points after treatment with eGFP mRNA@HbF (0.5 μg/mL). Data are presented as mean ± SEM (n=3) and were analyzed by one-way ANOVA followed by Tukey’s multiple comparisons test. Significance: ***p*≤0.01, *****p*≤0.0001.


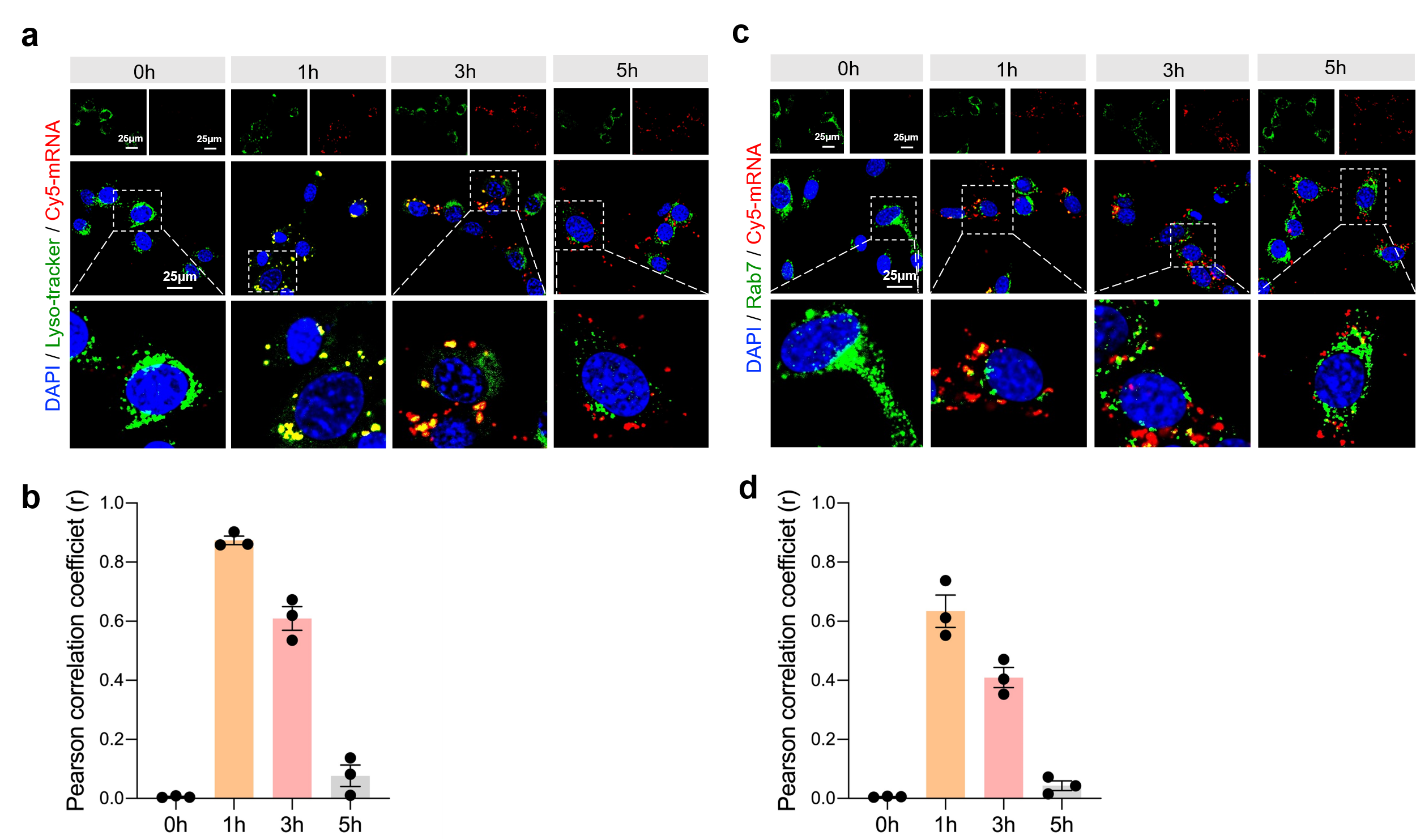


**Figure S16. Time-dependent endolysosomal escape of mRNA@HbF in mouse lung fibroblasts (MLFs).** (a, c) Representative images of MLFs incubated with Cy5-labeled mRNA@HbF (red) for the indicated times. Lysosomes and late endosomes were visualized using Lyso-tracker Green (a) and immunofluorescence against Rab7 (c), respectively; nuclei were counterstained with DAPI (blue). (b, d) Quantification of the co-localization presented as the Pearson correlation coefficient for panels (a) and (c). The high initial co-localization at 1 h progressively decreased over time, indicating efficient endosomal escape of the cargo. Data are mean ± SEM (n=3).


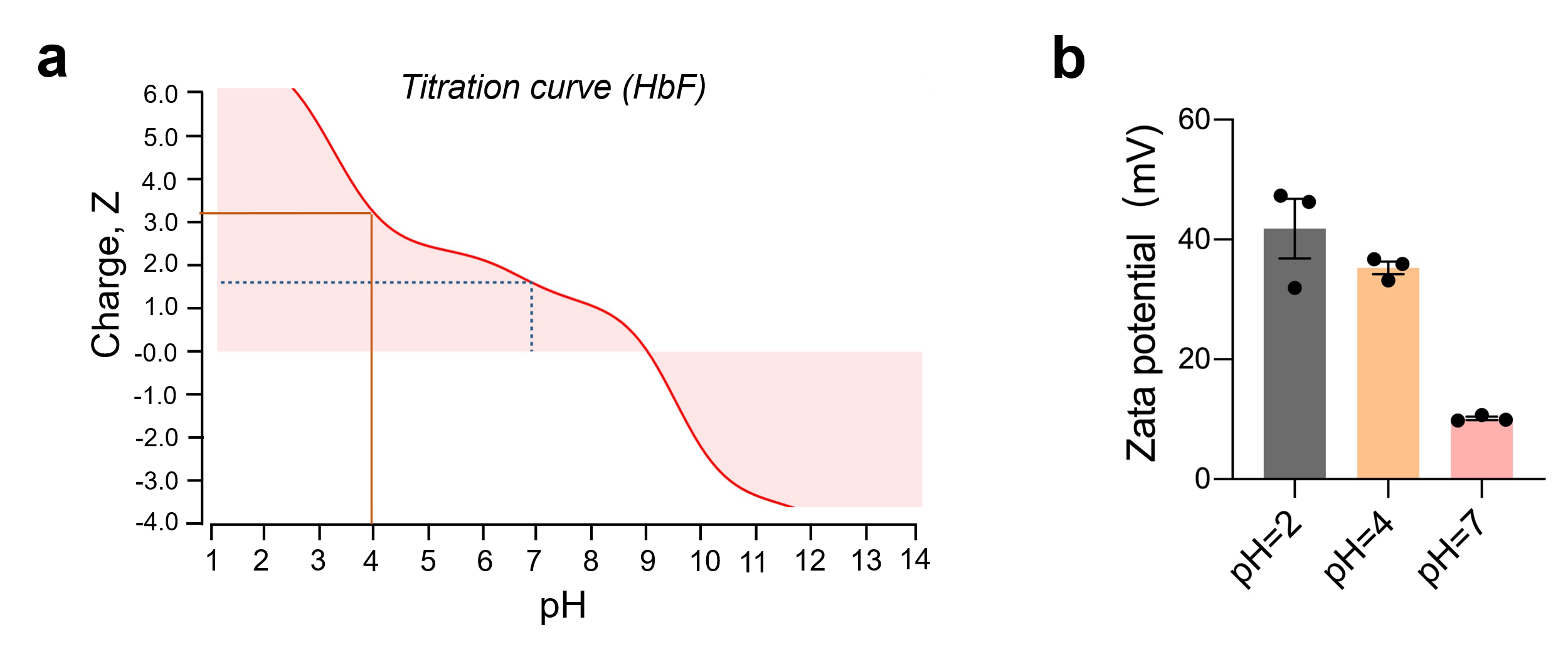


**Figure S17.** **Surface properties of HbFs support endosomal escape.** (a) Theoretical titration curve of HbFs. (b) Zeta potential measurement showing an increase in positive surface charge under acidic conditions, consistent with the protonation of surface‑exposed lysine and arginine residues. This charge reversal is critical for promoting endosomal membrane disruption and cargo release. Data are mean ± SEM (n=3).


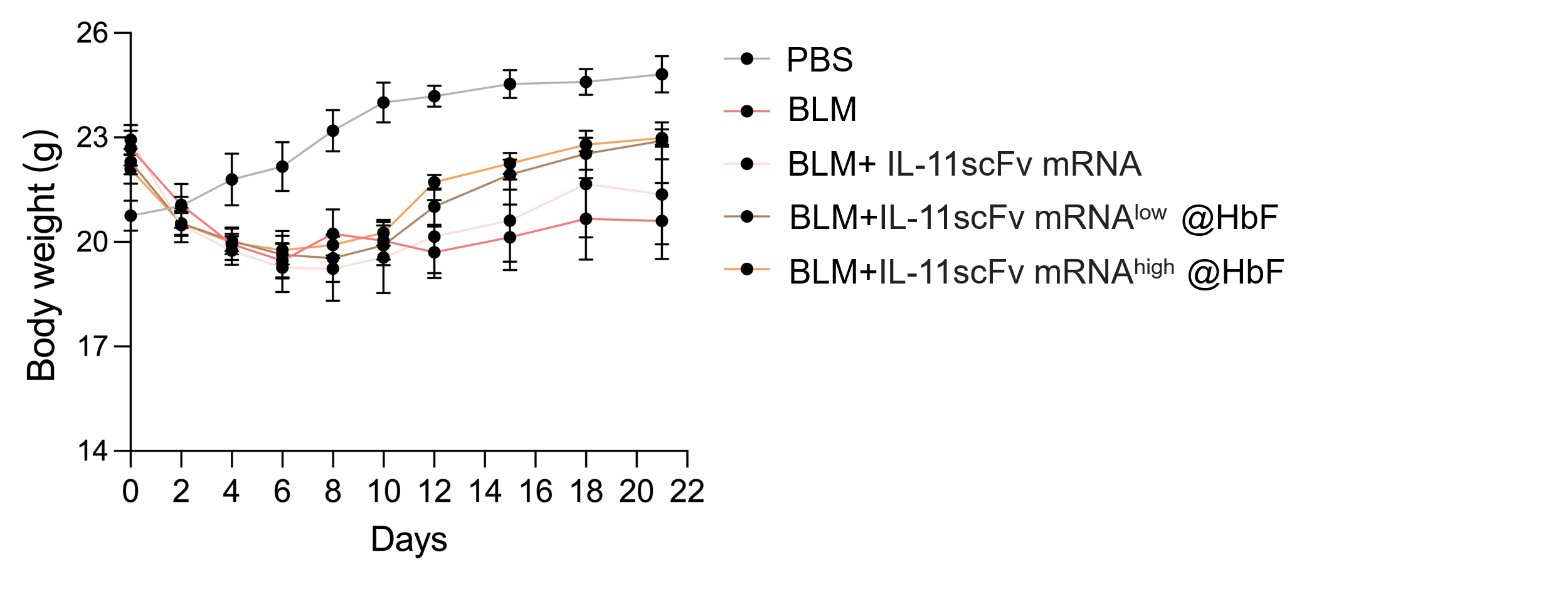


**Figure S18. Body weights of mice in different groups**; n=6.


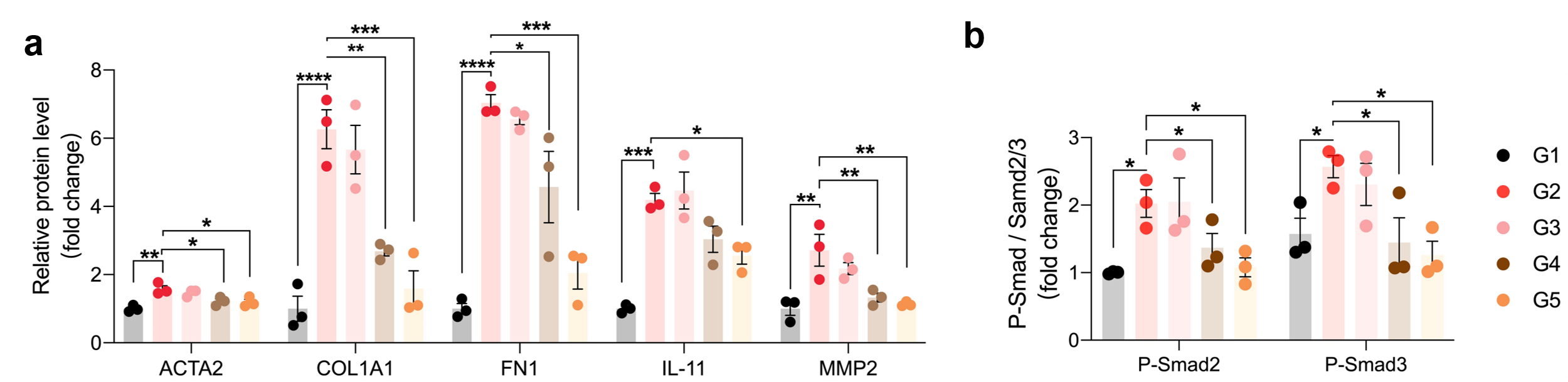


**Figure S19. Quantification of Western blot data from Figure 7i.** Data are presented as mean ± SEM (n=3) and were analyzed by one-way ANOVA followed by Tukey’s multiple comparisons test. Significance: **p*≤0.05, ***p*≤0.01, ****p*≤0.001, *****p*≤0.0001.

**
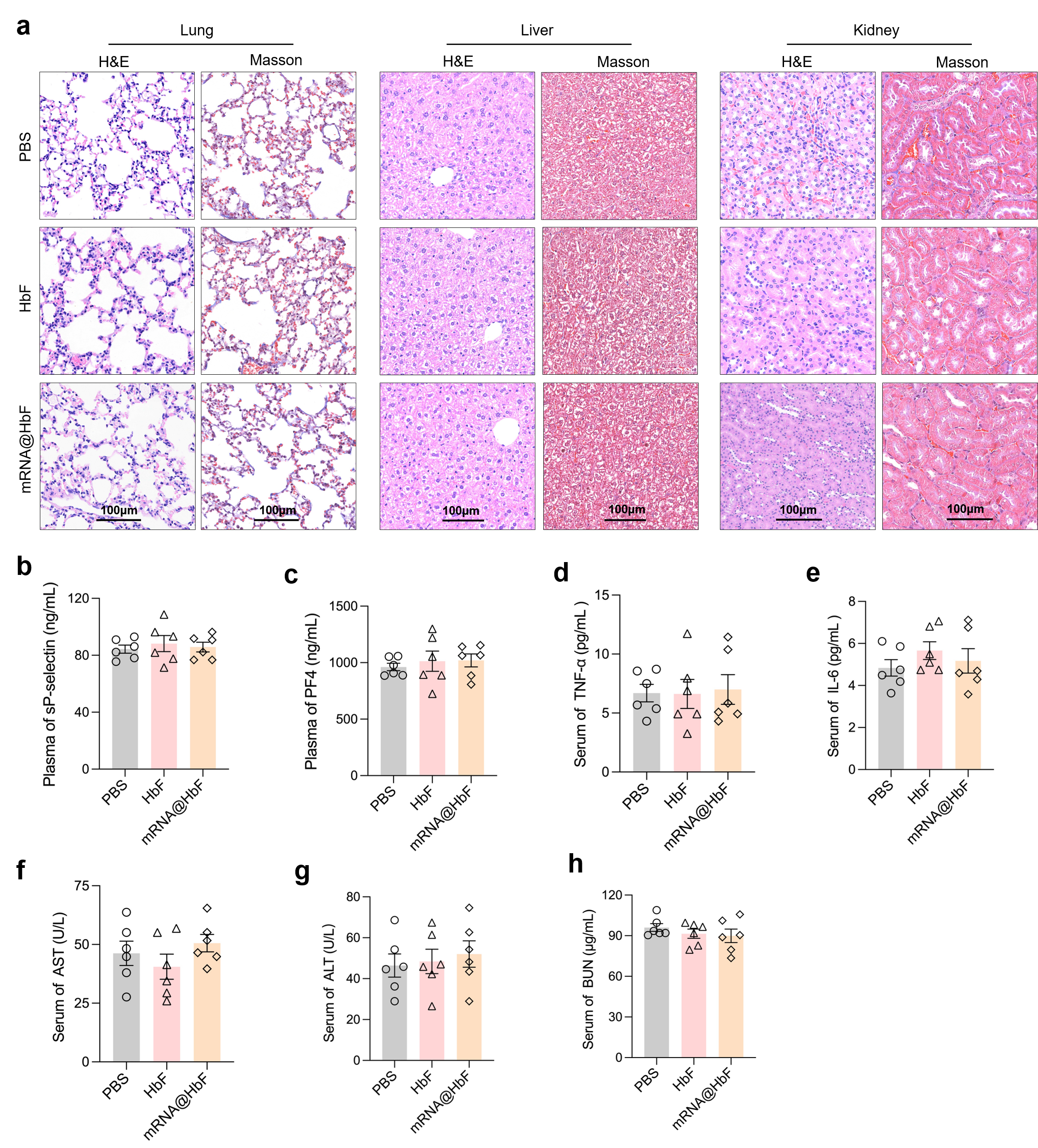
**

**Figure S20. In vivo safety evaluation after repeated intravenous administration of mRNA@HbFs.** Mice were treated with PBS, HbF (50 μg/mouse), or IL‑11 scFv mRNA@HbFs (50 μg HbF/mouse) every 3 days for 21 days. (a) Representative H&E and Masson’s trichrome staining images of lung, liver, and kidney sections. (b-c) Plasma sP-selectin and PF4 levels. (d-h) Serum TNF-α, IL-6, AST, ALT, and BUN levels. Data are presented as mean ± SEM (n=6) and were analyzed by one-way ANOVA followed by Tukey’s multiple comparisons test. No significant pathological alterations or abnormal biochemical changes were observed in the IL‑11 scFv mRNA@HbF-treated animals compared with controls.


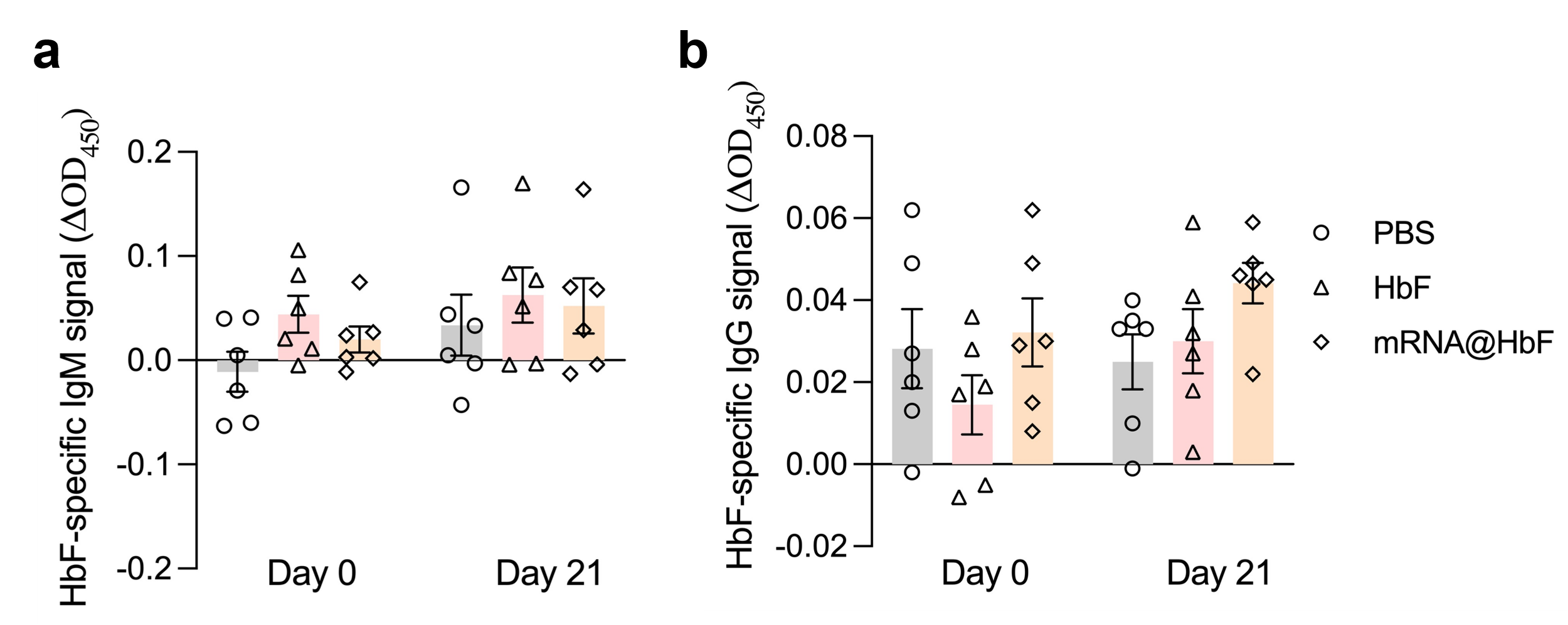


**Figure S21. Humoral immunogenicity evaluation of HbFs and mRNA@HbFs.** (a) HbF-specific IgM signal. (b) HbF-specific IgG signal. Using the same cohort of mice as in Fig. S20, serum samples were collected at day 0 and day 21 from the PBS, HbF, and mRNA@HbF groups. HbF-specific IgM and IgG responses were measured by indirect ELISA. Data are presented as mean ± SEM (n=6) and were analyzed by one-way ANOVA followed by Tukey’s multiple comparisons test.


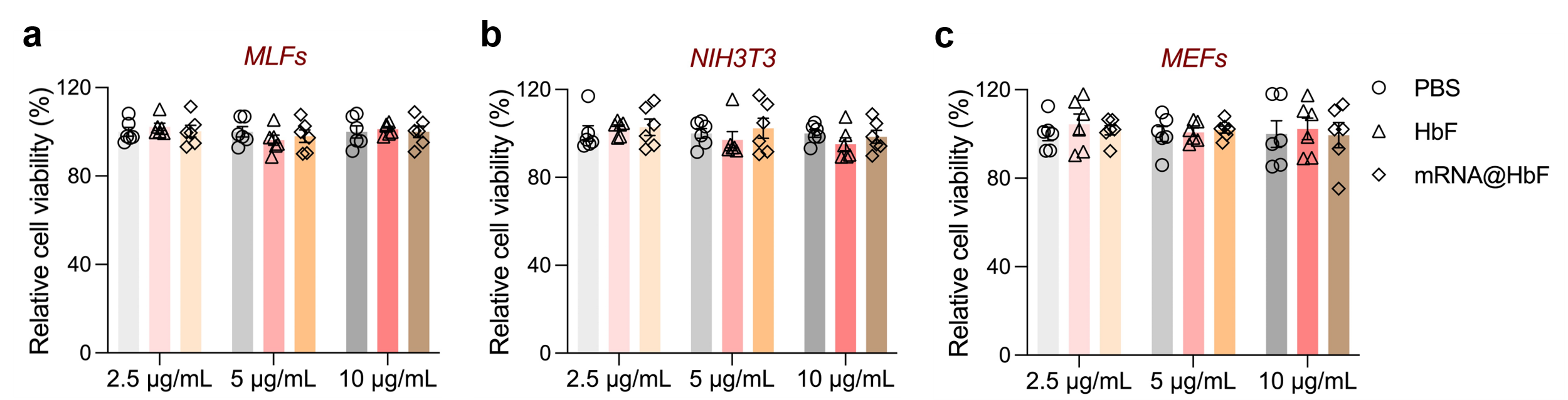


**Figure S22. CCK-8 assay of the effects of HbF and IL-11 scFv mRNA@HbF on cell viability.** (a) Primary mouse lung fibroblasts (MLFs), (b) NIH/3T3 cells, and (c) MEFs were treated with PBS, HbF, or IL-11 scFv mRNA@HbF for 24 h, and cell viability was measured by CCK-8. HbF was tested at 2.5, 5, and 10 µg/mL. The 2.5 µg/mL HbF dose corresponds to the amount of HbF present in IL-11 scFv mRNA@HbF at the working mRNA concentration used in the cellular assays (0.5 µg/mL). Data are presented as mean ± SEM (n=6) and were analyzed by one-way ANOVA followed by Tukey’s multiple comparisons test.


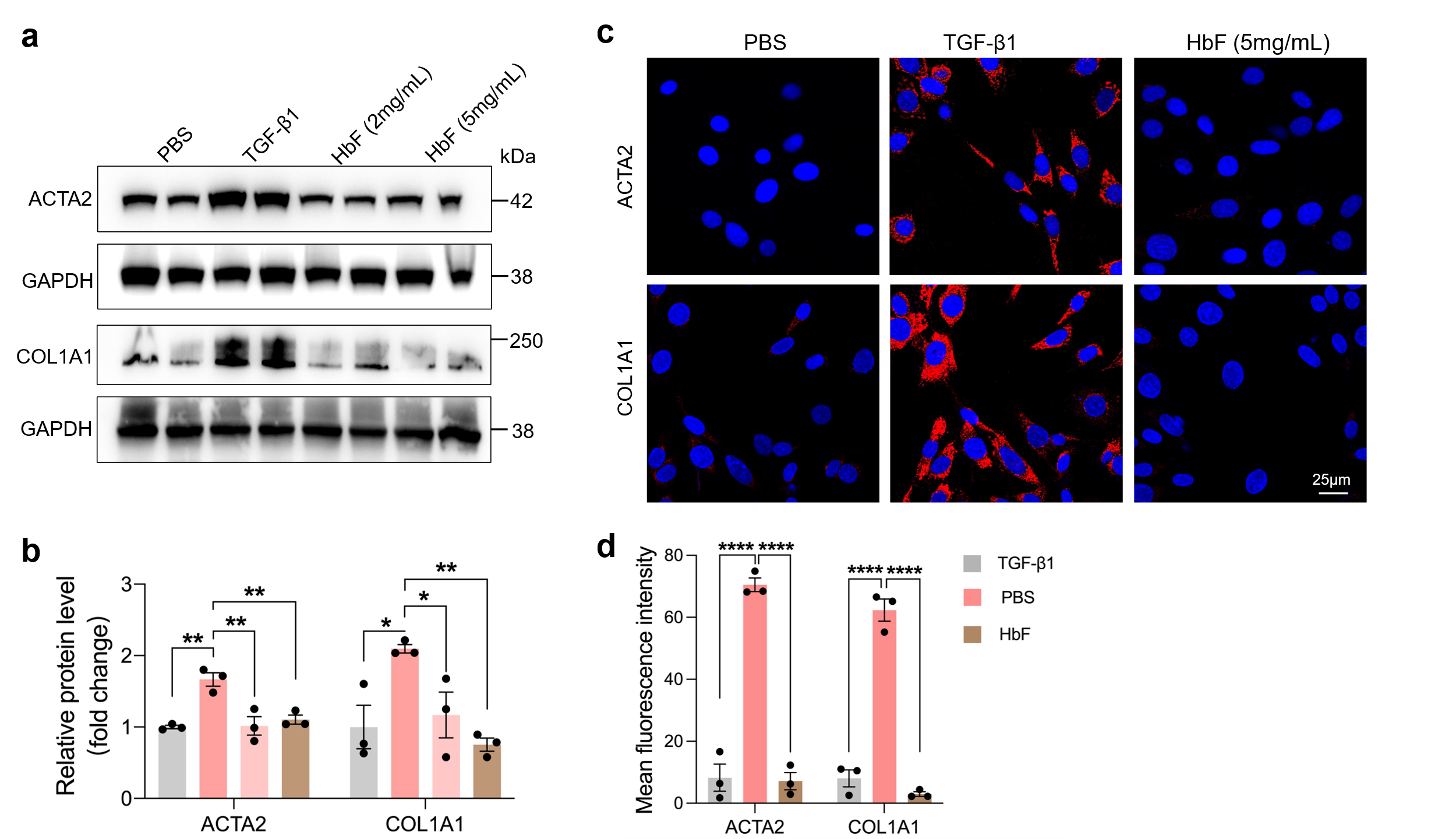


**Figure S23. HbF alone does not induce fibroblast activation in vitro.** (a) Representative Western blot and (b) quantitative analysis of fibroblast activation markers (ACTA2 and COL1A1) in NIH/3T3 cells treated for 24 h with PBS, TGF‑β1 (10 ng/mL; positive control), or HbF (2 and 5 mg/mL, 2.5 µL per 2 mL). HbF treatment did not upregulate these markers. Data are mean ± SEM (n=3) and were analyzed by one-way ANOVA followed by Tukey’s multiple comparisons test. Significance: **p* ≤ 0.05, ***p* ≤ 0.01, *****p* ≤ 0.0001.


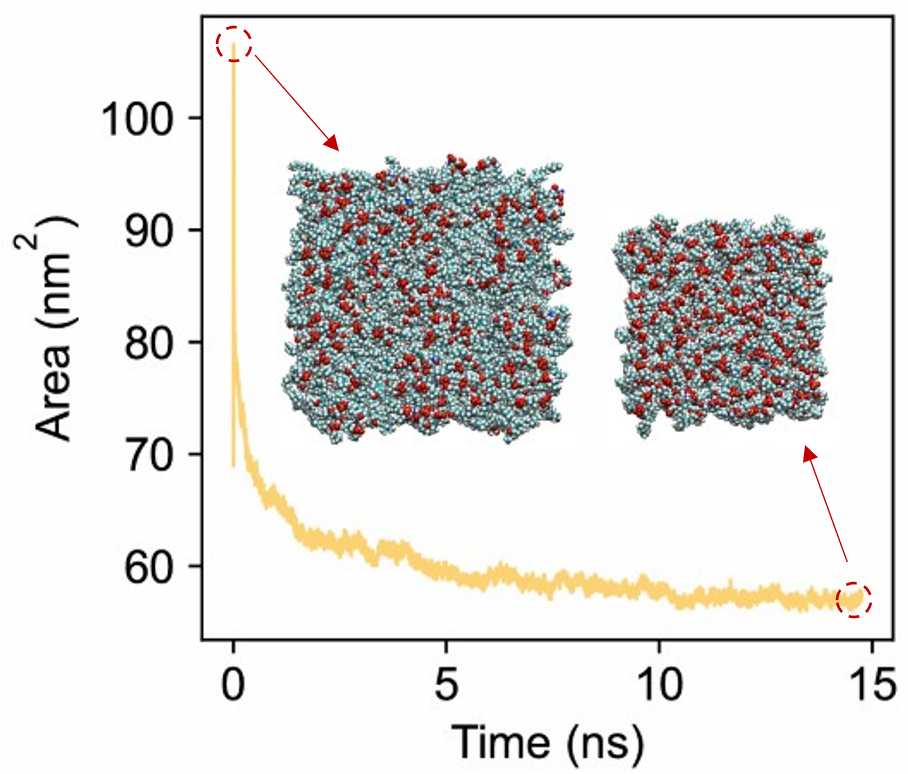


**Figure S24. Membrane system equilibration.** The simulated membrane reached equilibrium after 14.7 ns.

**Table S1. Cryo-EM data collection, refinement and validation statistics for PM1.**

|  | **Hb-PM1 (EMD-64995, PDB 9VE9)** |
| --- | --- |
| **Data collection and processing** | |
| Magnification | ×130,000 |
| Voltage (kV) | 300 |
| Electron exposure (e–/Å2) | 40 |
| Defocus range (μm) | -1.5 – -2.5 |
| Pixel size (Å) | 0.932 |
| Symmetry imposed | C1 |
| Helical rise (Å) | 4.782 |
| Helical twist (°) | -1.591 |
| Initial particle images (no.) | 1,215,070 |
| Final particle images (no.) | 31,408 |
| Map resolution (Å) | 3.3 |
| FSC threshold | 0.143 |
| Map resolution range (Å) | 200-3.3 |
| **Refinement** | |
| Initial model used (PDB code) | De novo |
| Model resolution (Å) | 3.5 |
| FSC threshold | 0.5 |
| Model resolution range (Å) | 200-3.5 |
| Map sharpening *B* factor (Å2) | 104 |
| Model composition | |
| Non-hydrogen atoms | 1,900 |
| Protein residues | 245 |
| Ligands | 0 |
| *B* factors (Å2) | |
| Protein | 61.59 |
| Ligand | – |
| R.m.s. deviations | |
| Bond lengths (Å) | 0.006 |
| Bond angles (°) | 1.117 |
| Validation | |
| MolProbity score | 2.18 |
| Clashscore | 6.84 |
| Poor rotamers (%) | 2.38 |
| Ramachandran plot | |
| Favored (%) | 91.11 |
| Allowed (%) | 8.89 |
| Disallowed (%) | 0 |

**Table S2. Composition of the asymmetric lipid bilayer model.**

| Lipid type | Upper leaflet | Lower leaflet |
| --- | --- | --- |
| Cholesterol | 36 | 24 |
| POPC | 54 | 24 |
| POPE | 0 | 36 |
| POPS | 6 | 24 |
| POPI | 0 | 12 |
| PSM | 24 | 0 |

**Table S3. Parameters for each molecular dynamics simulation system.**

| system | box size  a × b × c (nm^3^) | number of water molecules | ions | fibril orientation | simulation time (ns) |
| --- | --- | --- | --- | --- | --- |
| membrane | 8.3×8.3×8.5 | 9051 | 65 Na  23 Cl | / | 14.7 |
| membrane-fibril | 7.6×7.6×13 | 12193 | 22 Na | Direction 1 | 100 |
| membrane-fibril | 7.6×7.6×15 | 15413 | 22 Na | Direction 2 | 100 |
| membrane-fibril | 7.6×7.6×15 | 15426 | 22 Na | Direction 3 | 100 |
| membrane-fibril | 7.6×7.6×15 | 15397 | 22 Na | Direction 4 | 100 |


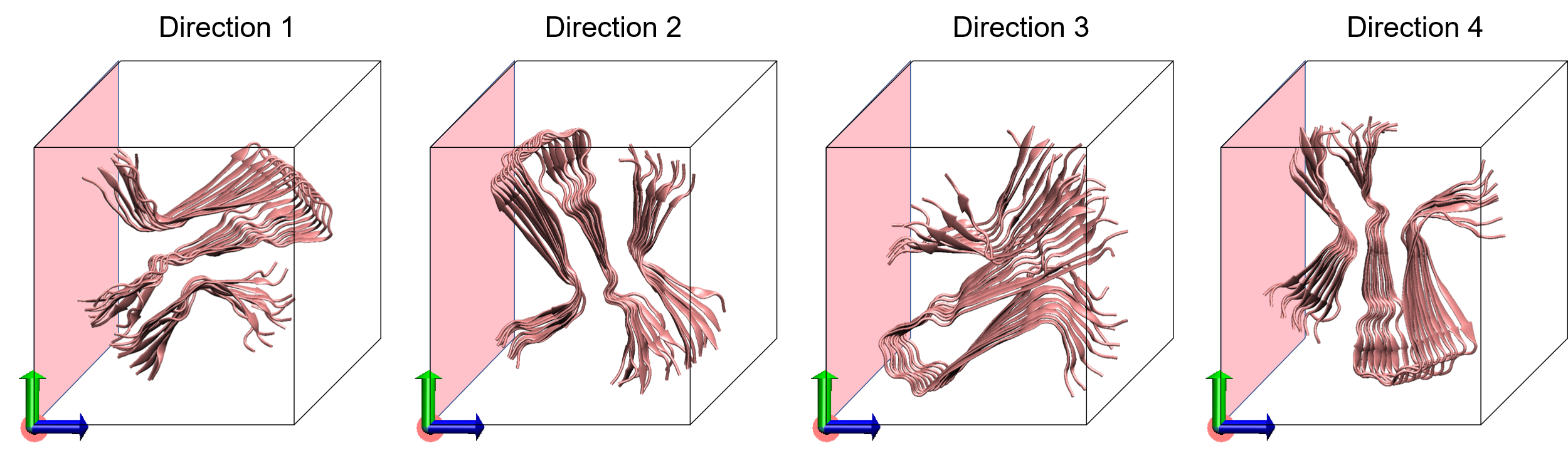


**Table S4. Amino acid and nucleotide sequences of IL-11 scFv.**

| **Amino acid sequence** |
| --- |
| **MGWSCIILFLVATATGVHS**MQVQLVQSGGGVVQPGRSLRLSCAASGFTFSSYGMHWVRQAPGKGLEWVAVISYDGSNKYYADSVKGRFTISRDNSKNTLYLQMNSLRAEDTAVYYCAKIGATDPLDYWGQGTLVTVSSGSASAPKLEEGEFSEARVQSALTQPRSVSGSPGQSVTLSCTGTSSDVGGYNYVSWYQHYPGKAPKLMIFDVNERSSGVPDRFSGSKSGNTASLTISGLQAEDEADYYCASYAGRYTWMFGGGTKVTVLGQPKAAPSVTLFPPSSGGSHHHHH* |
| **Nucleotide sequence** |
| **atgggctggagctgcatcatcctgttcctggtggccaccgccaccggcgtgcacagc**ATGCAAGTGCAGCTGGTGCAAAGCGGAGGCGGCGTGGTGCAACCCGGCAGATCCCTGAGGCTGAGCTGCGCCGCTAGCGGCTTCACCTTCAGCAGCTACGGCATGCACTGGGTGAGGCAAGCCCCTGGCAAGGGCCTGGAGTGGGTGGCCGTGATCAGCTACGACGGCAGCAACAAGTACTACGCCGACAGCGTGAAGGGAAGGTTCACCATCAGCAGAGACAACAGCAAGAACACCCTGTACCTGCAGATGAACGCCTGAGGGCCGAGGACACCGCCGTGTATACTGCGCTAAGATCGGCGCCACCGACCCTCTGGACTATTGGGGACAAGGCACCCTGGTGACAGTGTCCTCCGGCAGCGCTAGCGCCCCTAAGCTGGAGGAGGGCGAGTTTTCCGAGGCTAGGGTGCAGAGCGCTCTGACACAGCCTAGGTCCGTCTCCGGCAGCCCCGGACAATCCGTGACCCTGAGCTGCACCGGCACAAGCAGCGACGTGGGCGGCTACAACTACGTGAGCTGGTATCAGCACTACCCTGGCAAGGCTCCTAAGCTGATGATCTTCGACGTGAACGAAAGAAGCAGCGGCGTGCCTGATAGGTTCAGCGGCAGCAAGAGCGGCAACACCGCTAGCCTGACCATCAGCGGCCTGCAAGCCGAGGACGAGGCCGACTACTACTGCGCTAGCTACGCCGGAAGGTACACCTGGATGTTCGGCGGAGGCACCAGGTGACCGTGCTGGGACAGCCTAAGGCCGCCCCTAGCGTGACCCTGTTCCCTCCTAGCAGCGGCGGCAGCCACCATCACCATCACCACTGA |

**References**

[1] P.K. Dagur, J.P. McCoy Jr, Collection, storage, and preparation of human blood cells, *Current protocols in cytometry* 73, (2015): 5.1.1-5.1.16.

[2] Y. Feng, C. Liu, W. Cui, L. Yang, D. Wu, H. Zhang, X. Wang, Y. Sun, B. He, W. Dai, Engineering supramolecular peptide nanofibers for in vivo platelet-hitchhiking beyond ligand-receptor recognition, *Science Advances* 10, (2024): eadq2072.

[3] S.H.W. Scheres, RELION: Implementation of a Bayesian approach to cryo-EM structure determination, *Journal of Structural Biology* 180, (2012): 519-530.

[4] S.Q. Zheng, E. Palovcak, J.-P. Armache, K.A. Verba, Y. Cheng, D.A. Agard, MotionCor2: anisotropic correction of beam-induced motion for improved cryo-electron microscopy, *Nature Methods* 14, (2017): 331-332.

[5] A. Rohou, N. Grigorieff, CTFFIND4: Fast and accurate defocus estimation from electron micrographs, *Journal of Structural Biology* 192, (2015): 216-221.

[6] T. Bepler, A. Morin, M. Rapp, J. Brasch, L. Shapiro, A.J. Noble, B. Berger, Positive-unlabeled convolutional neural networks for particle picking in cryo-electron micrographs, *Nature Methods* 16, (2019): 1153-1160.

[7] P.V. Afonine, B.K. Poon, R.J. Read, O.V. Sobolev, T.C. Terwilliger, A. Urzhumtsev, P.D. Adams, Real-space refinement in PHENIX for cryo-EM and crystallography, *Acta Crystallographica Section D* 74, (2018): 531-544.

[8] K. Jamali, L. Käll, R. Zhang, A. Brown, D. Kimanius, S.H.W. Scheres, Automated model building and protein identification in cryo-EM maps, *Nature* 628, (2024): 450-457.

[9] P. Emsley, B. Lohkamp, W.G. Scott, K. Cowtan, Features and development of Coot, *Acta Crystallographica Section D* 66, (2010): 486-501.

[10] V.B. Chen, W.B. Arendall, III, J.J. Headd, D.A. Keedy, R.M. Immormino, G.J. Kapral, L.W. Murray, J.S. Richardson, D.C. Richardson, MolProbity: all-atom structure validation for macromolecular crystallography, *Acta Crystallographica Section D* 66, (2010): 12-21.

[11] E.L. Wu, X. Cheng, S. Jo, H. Rui, K.C. Song, E.M. Dávila-Contreras, Y. Qi, J. Lee, V. Monje-Galvan, R.M. Venable, J.B. Klauda, W. Im, CHARMM-GUI Membrane Builder toward realistic biological membrane simulations, *Journal of Computational Chemistry* 35, (2014): 1997-2004.

[12] W. Humphrey, A. Dalke, K. Schulten, VMD: Visual molecular dynamics, *Journal of Molecular Graphics* 14, (1996): 33-38.

[13] J. Huang, A.D. MacKerell Jr, CHARMM36 all-atom additive protein force field: Validation based on comparison to NMR data, *Journal of Computational Chemistry* 34, (2013): 2135-2145.

[14] S. Li, S. Li, Y. Cheng, Y. Fang, Q. Cao, Y. Cao, Dual Hydrophilic-Hydrophobic Core Architecture in Soy Glycinin Amyloid Fibrils Revealed by Cryo-EM, *Advanced Science* 12, (2025): e09821.

[15] T. Ashcroft, J.M. Simpson, V. Timbrell, Simple method of estimating severity of pulmonary fibrosis on a numerical scale, *Journal of clinical pathology* 41, (1988): 467-470.
